# Exploring the Complexity of Protein-Level Dosage Compensation that Fine-Tunes Stoichiometry of Multiprotein Complexes

**DOI:** 10.1371/journal.pgen.1009091

**Published:** 2020-10-28

**Authors:** Koji Ishikawa, Akari Ishihara, Hisao Moriya

**Affiliations:** 1 Research Core for Interdisciplinary Sciences, Okayama University, Okayama, Japan; 2 Course of Agrochemical Bioscience, Faculty of Agriculture, Okayama University, Okayama, Japan; Columbia University, UNITED STATES

## Abstract

Proper control of gene expression levels upon various perturbations is a fundamental aspect of cellular robustness. Protein-level dosage compensation is one mechanism buffering perturbations to stoichiometry of multiprotein complexes through accelerated proteolysis of unassembled subunits. Although N-terminal acetylation- and ubiquitin-mediated proteasomal degradation by the Ac/N-end rule pathway enables selective compensation of excess subunits, it is unclear how widespread this pathway contributes to stoichiometry control. Here we report that dosage compensation depends only partially on the Ac/N-end rule pathway. Our analysis of genetic interactions between 18 subunits and 12 quality control factors in budding yeast demonstrated that multiple E3 ubiquitin ligases and N-acetyltransferases are involved in dosage compensation. We find that N-acetyltransferases-mediated compensation is not simply predictable from N-terminal sequence despite their sequence specificity for N-acetylation. We also find that the compensation of Pop3 and Bet4 is due in large part to a minor N-acetyltransferase NatD. Furthermore, canonical NatD substrates histone H2A/H4 were compensated even in its absence, suggesting N-acetylation-independent stoichiometry control. Our study reveals the complexity and robustness of the stoichiometry control system.

## Author summary

Quality control of multiprotein complexes is important for maintaining homeostasis in cellular systems that are based on functional complexes. Proper stoichiometry of multiprotein complexes is achieved by the balance between protein synthesis and degradation. Recent studies showed that translation efficiency tends to scale with stoichiometry of their subunits. On the other hand, although protein N-terminal acetylation- and ubiquitin-mediated proteolysis pathway is involved in selective degradation of excess subunits, it is unclear how widespread this pathway contributes to stoichiometry control due to the lack of a systematic investigation using endogenous proteins. To better understand the landscape of the stoichiometry control system, we examined genetic interactions between 18 subunits and 12 quality control factors (E3 ubiquitin ligases and N-acetyltransferases), in total 114 combinations. Our data suggest that N-acetyltransferases are partially responsible for stoichiometry control and that N-acetylation-independent pathway is also involved in selective degradation of excess subunits. Therefore, this study reveals the complexity and robustness of the stoichiometry control system. Further dissection of this complexity will help to understand the mechanisms buffering gene expression perturbations and shaping proteome stoichiometry.

## Introduction

Controlling intracellular protein concentration at the proper level is a critical aspect of cellular systems. For example, it has been suggested that the cells regulate proteome concentration for a constant rate of biochemical reactions [1–3]. Moreover, since a wide variety of stress response pathways dynamically change the expression of proteins responsible for survival in various challenging environments, it is obvious that the control of protein concentration is important for coping with perturbations and maintaining cellular homeostasis.

Genome-wide studies measuring cellular robustness against genetic perturbations showed that *Saccharomyces cerevisiae* cells are fragile against protein overexpression of a subset of the genome [4, 5]. This finding indicates that the cell system is generally robust against genetic perturbations to various biological processes. However, it is unclear how cells buffer such environmental changes [6]. We recently reported that upon an increase in gene copy number, approximately 10% of the yeast genome are transcribed linearly into mRNA levels but not directly translated into protein levels [7]. Comprehensive analyses of aneuploid yeast and mammalian cells also showed that approximately 20% of the genome exhibit this molecular phenotype [8, 9]. Therefore, this phenomenon known as protein-level dosage compensation may partially explain the buffering of gene expression perturbations.

Protein-level dosage compensation predominantly targets genes encoding subunits of multiprotein complexes [7–10]. The concentration of the dosage-compensated proteins bidirectionally changes in response to that of its partner subunits [7, 11]. These observations are consistent with a hypothesis predicting the deleterious effects due to stoichiometric imbalance of the complex subunits on cell growth [5, 6, 12]. Another line of evidence for this prediction comes from ribosome profiling analyses revealing a proportional synthesis strategy that enables translation efficiency to scale with subunit stoichiometry [13, 14]. This finding shows that stoichiometric balance of multiprotein complex subunits is mainly maintained at the translational level. In addition, this strategy is conserved from bacteria to higher eukaryotes [14]. In this context, dosage compensation can be recognized as a fail-safe mechanism that fine-tunes proteome stoichiometry [15].

**Fig 1:**
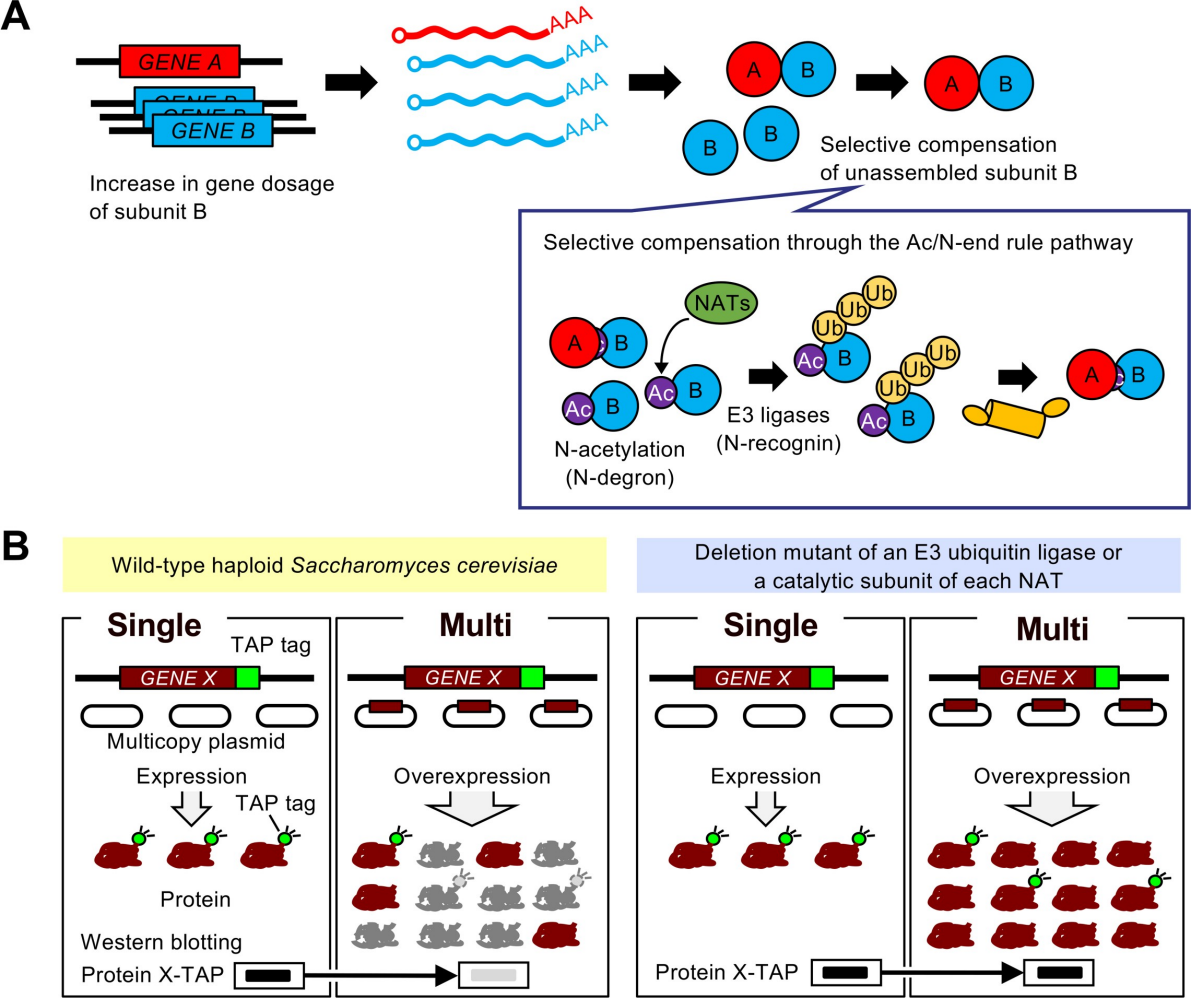
Genetic perturbation analysis of the stoichiometry control system. (**A**) Protein-level dosage compensation predominantly targets subunits of multiprotein complexes. The Ac/N-end rule pathway is involved in the underlying mechanism for the selective compensation of unassembled subunits. (**B**) An experimental setup for measuring the contribution of each quality control factor to dosage compensation. The untagged dosage-compensated protein was expressed from multicopy plasmid pTOW40836 containing the native regulatory sequences, including promoter and 5´ and 3´ untranslated regions. If the target gene is subjected to dosage compensation, the level of the TAP-tagged target protein expressed only from the chromosome decreases upon an increase in gene copy number (Multi) compared to vector control (Single). The Multi/Single ratio is higher than that in WT cells in the deletion mutant of a gene encoding an E3 ubiquitin ligase or a catalytic subunit of each NAT, if the deleted factor contributes to the compensation of the target gene.

The mechanism of dosage compensation is accelerated degradation of unassembled subunits by the ubiquitin–proteasome system [7, 11, 16, 17]. Multiple E3 ubiquitin ligases involved in stoichiometry control were previously identified [11, 18, 19], but we lack an understanding of their relative contribution to the compensation of identical subunits [20]. Furthermore, the underlying mechanism for selective degradation of excess subunits is not well understood. Although previous studies found evidence for selective compensation of Cog1 and Hcn1 in yeast and PLIN2 in mammalian cells by the Ac/N-end rule pathway [11, 21], it is unclear how widespread this pathway contributes to the compensation. The Ac/N-end rule pathway is one mechanism for protein degradation through the acetylation of N-terminal amino acid by N-acetyltransferases (NATs). With the limited number of examples, N-acetylation was shown to act as a degradation signal (N-degron) recognized by specific E3 ubiquitin ligases (N-recognin) [11, 18, 22]. It has been hypothesized that N-degron of unassembled subunits is exposed and recognized by N-recognin, whereas that of assembled subunit is shielded by the other subunits in the same complex and is inaccessible by N-recognin (Fig 1A) [11, 18, 23]. This model is consistent with the biological role of dosage compensation, but it remains to be investigated whether the compensation depends on this pathway. Furthermore, the impact of the Ac/N-end rule pathway on stoichiometry control differs between perturbed and unperturbed conditions [11, 18, 24], which prompted us to estimate how widespread NATs-mediated compensation occurs upon genetic perturbations.

In this study, we measured the contribution of E3 ubiquitin ligases and NATs to dosage compensation of the RNase P/MRP subunits, which are the compensated proteins identified in our previous study [7], in a systematic manner. We find that multiple E3 ubiquitin ligases and NATs are involved in the compensation. We also find that the dependency of the compensation on NATs is variable among tested subunits. Our findings suggest that dosage compensation does not completely depend on the Ac/N-end rule pathway and highlight the complexity of the stoichiometry control system.

## Results

### Multiple E3 ubiquitin ligases Tom1 and Not4 are involved in dosage compensation of identical subunits

Our previous study screened 54 genes on chromosome I of *S*. *cerevisiae* in order to unbiasedly estimate how much of the genome encodes the compensated proteins, and we identified five proteins (Rbg1, Mtw1, Pop5, Saw1, and Erp2) [7]. To investigate which E3 ubiquitin ligases are involved in dosage compensation and their relative contribution, we examined whether these proteins are less compensated in five deletion mutants of E3 ubiquitin ligase genes (*DOA10*, *NOT4*, *TOM1*, *SAN1*, and *UBR1*) involved in the protein quality control system including the N-end rule pathways: (i) Doa10 and Not4 are known as N-recognins in the Ac/N-end rule pathway [11, 18], (ii) Tom1 is involved in degradation of a broad range of unassembled ribosomal subunits [19], (iii) San1 is responsible for proteasomal degradation of aberrant nuclear proteins [25], and (iv) Ubr1 is N-recognin in the Arg/N-end rule pathway that is suggested to complement the Ac/N-end rule pathway [26, 27]. Using a previously developed method for identifying genes with dosage compensation (S1A Fig) [7], we found that the amount of Pop5 in *tom1*Δ and *not4*Δ and Saw1 in *doa10*Δ was increased compared to wild-type (WT) cells (S1B and S1C Fig). A recent systematic analysis of protein stability in *doa10*Δ cells showed that the hydrophobicity of N-terminus negatively correlates with the protein stability [24]. Consistent with this finding, N-terminus of Saw1 is more hydrophobic than the other proteins that are not stabilized in *doa10*Δ cells. However, we note that Pop5 with a hydrophobic N-terminus was not stabilized in the absence of Doa10.

Pop5 is a subunit of the RNase P/MRP complexes comprising of eleven subunits [28]. These complexes share eight subunits: Pop1, Pop3, Pop4, Pop5, Pop6, Pop7, Pop8, and Rpp1, while Rpr2 is included only in the RNase P and Snm1 and Rmp1 are included only in the RNase MRP. We previously found that translation efficiency of *POP5* mRNA does not change upon an increase in *POP5* copy number by ribosome profiling [7]. In addition, translation efficiency of the other subunits of the RNase P/MRP complexes, including Rpp1 and Pop8 which form a subcomplex with Pop5 [29], is also not affected in the same condition. Therefore, stoichiometry control of these complexes depends on protein degradation upon genetic perturbations, and it is possible to more precisely assess its contribution to stoichiometry control by analyzing the RNase P/MRP subunits.

**Fig 2:**
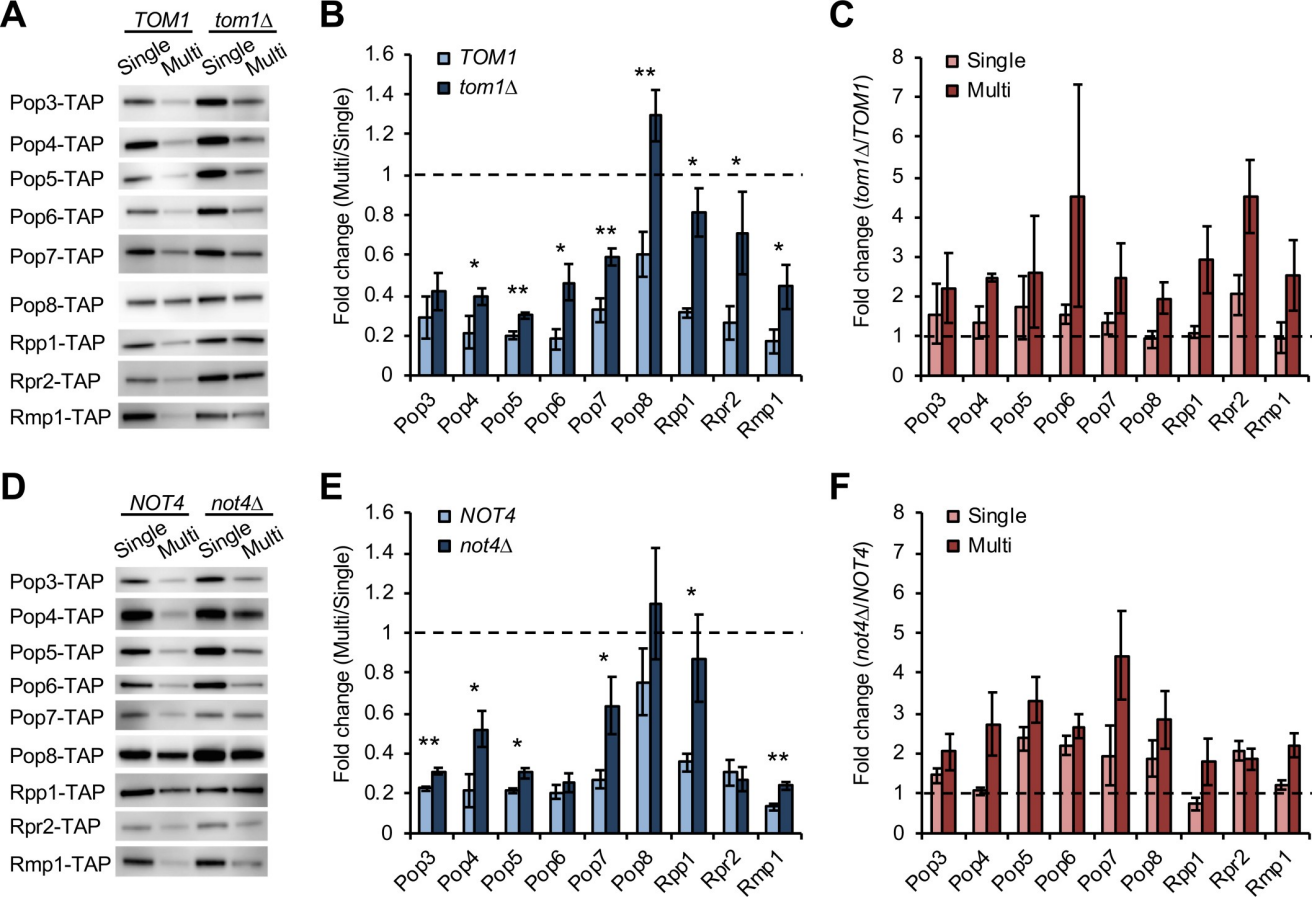
Tom1 and Not4 are involved in dosage compensation. (**A**) Western blots of the RNase P/MRP subunits in *tom1*Δ cells. The TAP-tagged subunit expressed from the chromosome was detected with PAP. Pop1 and Snm1 were not detected in our experiments. A representative blot from three biological replicates is shown. (**B**) Quantification of protein levels in the Multi condition relative to those in the Single condition in WT and *tom1*Δ cells. The average fold change ± s.d. was calculated from three biological replicates. Statistical significance was determined by a two-tailed Welch’s *t* test (*p<0.05, **p<0.01). Dashed line represents the same expression level between the Single and Multi conditions. (**C**) Comparison of protein levels between WT and *tom1*Δ cells in the Single or Multi conditions. The average fold change ± s.d. was calculated from three biological replicates. Dashed line represents the same expression level between WT and *tom1*Δ cells. Pop6 and Rpr2 levels were marginally significantly increased in *tom1*Δ under the Single condition (*p* = 0.059 and 0.071, respectively). (**D–F**) Same as in (**A–C**), except that shown are Western blots of the RNase P/MRP subunits in *not4*Δ cells (**D**) and their quantification (**E, F**). Pop5, Pop6, and Rpr2 levels were significantly increased in *not4*Δ compared to WT cells under the Single condition (*p*<0.05).

Since most subunits of these complexes are subjected to dosage compensation [7], we examined whether Tom1 and Not4 are involved in the compensation of the other subunits. We used an experimental setup by which the TAP-tagged target protein expressed from only one genomic locus is detected upon an increase in copy number of untagged version of the same gene (Fig 1B and S2A–S2D Fig). We first compared protein levels between with and without overexpression in WT, *tom1*Δ, or *not4*Δ cells (S2E Fig). This analysis showed a significant decrease in the compensation of Pop4, Pop5, Pop6, Pop7, Pop8, Rpp1, Rpr2, and Rmp1 in *tom1*Δ cells (Fig 2A and 2B), as well as Pop3, Pop4, Pop5, Pop7, Rpp1, and Rmp1 in *not4*Δ cells (Fig 2D and 2E). Therefore, Tom1 and Not4 were involved in dosage compensation of the RNase P/MRP subunits: Tom1 and Not4 target at least 8 and 6 out of 11 subunits, respectively (Table 1).

**Table 1:** Responsible E3 ubiquitin ligases and NATs for dosage compensation of the RNase P/MRP subunits.

Subunit	N-terminal first five amino acids	Responsible E3 (Tom1 or Not4)	Responsible NATs (Sequence-based)	Responsible NATs (Experiment-based)
Pop1	MSGSL	n.d.	NatA	n.d.
Pop3	MSGSL	Not4	NatA	NatD
Pop4	MDRTQ	Tom1, Not4	NatB	NatA
Pop5	MVRLK	Tom1, Not4	NatA	NatA^a^
Pop6	MINGV	Tom1	NatC	NatB
Pop7	MALKK	Tom1, Not4	NatA	not identified
Pop8	MGKKT	Tom1	NatA	NatB
Rpp1	MLVDL	Tom1, Not4	NatC	not identified
Rpr2	MGKKA	Tom1	NatA	NatB
Snm1	MNKDQ	n.d.	NatB	n.d.
Rmp1	MDEMD	Tom1, Not4	NatB	NatA, NatB

a Pop5 was significantly more compensated in *naa10*Δ than in WT cells. n.d.: not detected

We further examined whether Doa10 is also involved in stoichiometry control of the RNase P/MRP subunits and found that all nine tested subunits were compensated in *doa10*Δ to the same degree in WT cells (S1 and S3 Figs). Therefore, Doa10 is not involved in stoichiometry control of the RNase P/MRP subunits, consistent with a previous study showing that Cog1 is compensated by Not4 instead of Doa10 [11]. These observations suggest different substrate preferences of E3 ubiquitin ligases responsible for the compensation. Indeed, Tom1 tends to compensate nuclear-localized proteins, as described below (see Discussion).

We next compared protein levels between WT and *tom1*Δ or *not4*Δ cells with or without overexpression of each subunit of the RNase P/MRP complexes (S2F Fig). This analysis showed the higher level of Pop6 and Rpr2 in *tom1*Δ and Pop5, Pop6, and Rpr2 in *not4*Δ than those in WT cells without an increase in gene copy number (Fig 2C and 2F, Single). As this comparison is between vector controls in WT and mutant cells, Tom1 and Not4 may be involved in degradation of these subunits in unperturbed conditions. In addition, since Pop6 was not subjected to Not4-mediated compensation, Not4 contributes to its degradation but this is not accelerated upon overexpression.

Our analysis showed that Not4 contributes to the compensation of Rmp1 whose polylysine sequence CKKKKKRKKKNK, close to the C-terminal (residues 182–193), induces translation arrest coupled with Not4-mediated proteolysis [30]. Recent studies revealed that translation arrest peptides are degraded by the ribosome quality control complex (RQC) [31]. The RQC-mediated degradation of arrest peptides and the initiation of this pathway are mediated by E3 ubiquitin ligases Ltn1 and Hel2, respectively [32, 33]. To examine whether this clearance pathway for arrest peptides is linked to dosage compensation, we measured the compensation degree of Rmp1 in deletion mutants of *LTN1* or *HEL2* using the same experimental setup shown in Fig 1B. We found that the compensation degree of Rmp1 in *ltn1*Δ and *hel2*Δ was compatible with that in WT cells (S4 Fig). Our results suggest that perturbations to a quality control pathway for the clearance of arrest peptides does not result in a decreased level of Rmp1 compensation.

### Dosage compensation of Pop6 and Pop7 occurs in response to their intracellular concentration

**Fig 3:**
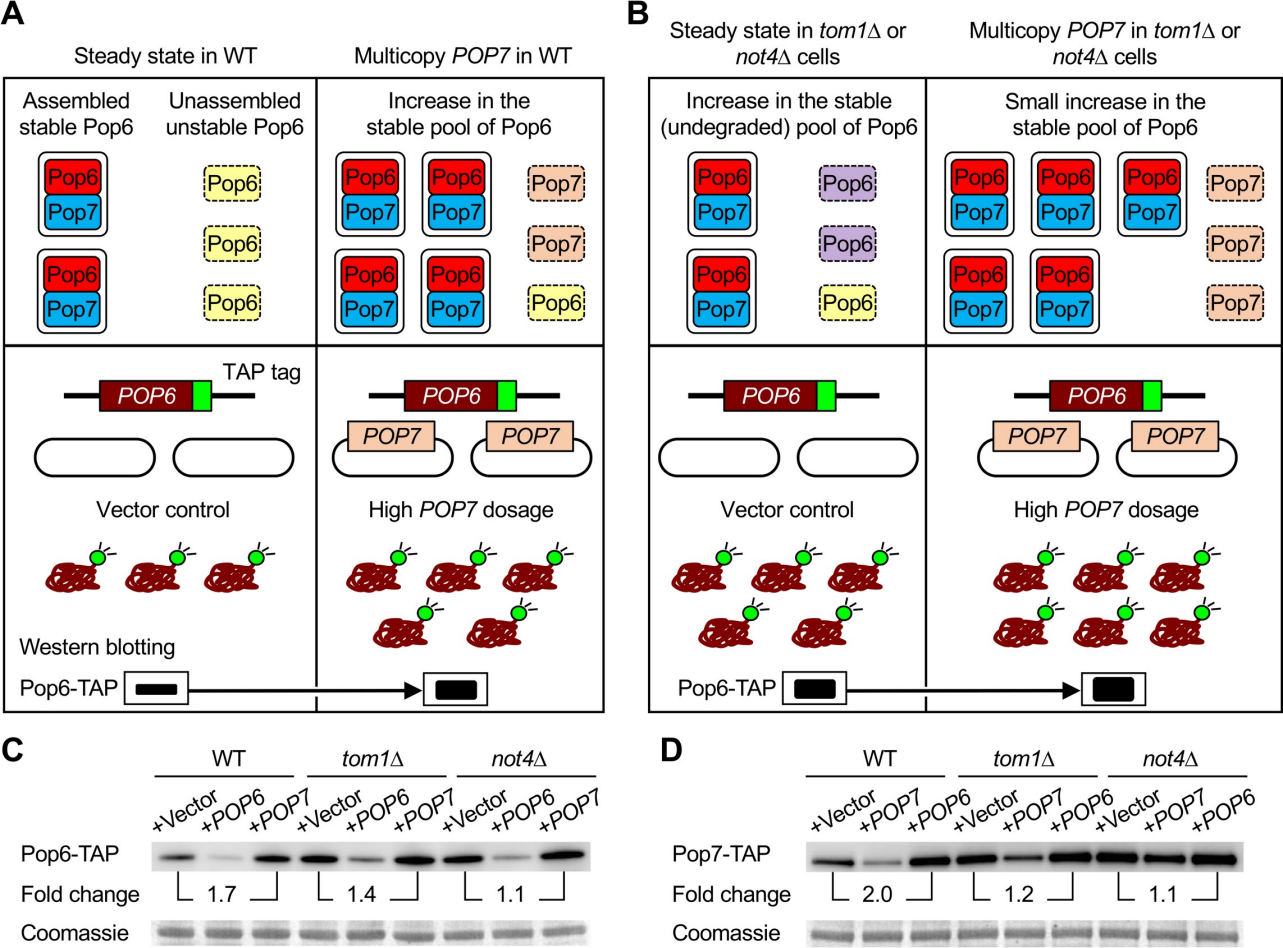
Bidirectional dosage compensation of the Pop6–Pop7 heterodimeric subcomplex. (**A**) A model for stoichiometry control of Pop6 and Pop7 in WT cells (top) and an experimental setup for testing this model (bottom). Pop6 becomes more stable when in complex with the partner subunit Pop7, while unassembled Pop6 is less stable (top left). Upon multicopy expression of Pop7, potentially degraded Pop6 becomes stable by forming the complex with excess Pop7 (top right). Pop6 level in vector control is the steady state level (bottom left). If Pop6 turnover is reduced by high *POP7* dosage, Pop6 level is increased compared to that in vector control (bottom right). (**B**) Same as in (**A**), except that shown is the case of *tom1*Δ or *not4*Δ cells. Potentially degraded Pop6 in WT cells becomes stable in the absence of Tom1 or Not4 that are responsible for Pop6 and Pop7 degradation (top left). Upon multicopy expression of Pop7, this undegraded fraction and the remaining unstable pool of Pop6 become stable by forming the complex with excess Pop7 (top right). The steady state level of Pop6 in *tom1*Δ or *not4*Δ cells is higher than that in WT cells (bottom left). If the turnover of Pop6 is further reduced by high *POP7* dosage, it leads to a small increase in Pop6 level (bottom right). (**C, D**) Western blots of Pop6-TAP (**C**) and Pop7-TAP (**D**) in WT, *tom1*Δ, and *not4*Δ cells. The TAP-tagged proteins were detected with PAP in the Single (+Vector: pTOW40836) and Multi (+*POP6*: pTOW40836-*POP6*, +*POP7*: pTOW40836-*POP7*) conditions. Quantification of band intensities relative to the Single condition in each strain is shown. Coomassie staining of a 50-kDa protein, corresponding to enolase, is shown as a loading control.

Since both Pop6 and Pop7, which form the Pop6–Pop7 heterodimeric subcomplex [34, 35], are subjected to dosage compensation (Fig 2), the amount of Pop6 and Pop7 may change in response to that of partner subunits. We hypothesized that high *POP7* dosage leads to changes in the fraction of the stable/assembled and unstable/unassembled pools of Pop6 (Fig 3A, top). Furthermore, since the mechanism of dosage compensation is accelerated degradation of unassembled subunits, the balance of these subunit pools may be perturbed in the absence of Tom1 or Not4 (Fig 3B, top). As expected (Fig 3A and 3B, bottom), we found that Pop6 was stabilized by an increase in *POP7* dosage, and vice versa, in WT, *tom1*Δ, or *not4*Δ cells (Fig 3C and 3D). These results indicate that dosage compensation of Pop6 and Pop7 occurs bidirectionally in response to changes in intracellular concentration of each subunit: accelerated degradation of Pop6/Pop7 upon high *POP6*/*POP7* dosage (downward compensation) and reduced degradation of Pop6/Pop7 upon high *POP7*/*POP6* dosage (upward compensation).

Upon increasing gene copy number of the partner subunit, the relative ratio of Pop6 and Pop7 in *tom1*Δ and *not4*Δ was lower than that in WT cells. This difference may reflect the regulation of the compensation degree, as the amount of Pop6 and Pop7 was increased in both mutants without changes in gene copy number (Fig 3C and 3D, +Vector). Therefore, Tom1 and Not4 impact the balance of stable/unstable subunit pools of the Pop6–Pop7 heterodimeric subcomplex.

### NATs contribute to dosage compensation in a complex manner

**Fig 4:**
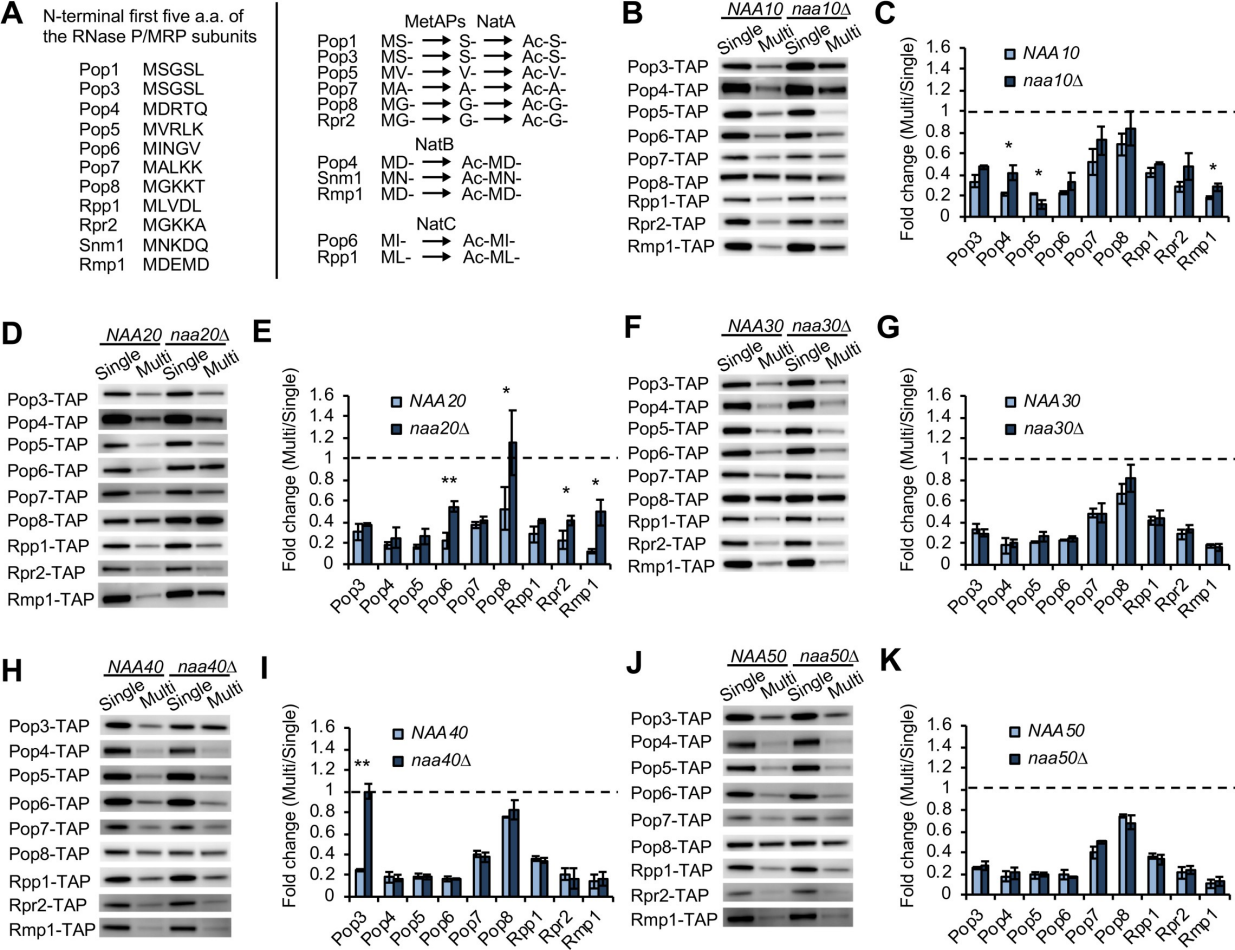
NATs contribute to dosage compensation in a complex manner. (**A**) The first five amino acids of the RNase P/MRP subunits (left). Based on these sequences, NatA, NatB, or NatC are responsible for their N-acetylation (right). NatA-mediated acetylation requires removal of N-terminal Methionine by Met-aminopeptidases Map1 and Map2 (MetAPs). (**B**) Western blots of the RNase P/MRP subunits in *naa10*Δ cells. The TAP-tagged subunit expressed from the chromosome was detected with PAP. Pop1 and Snm1 were not detected in our experiments. A representative blot from three biological replicates is shown. (**C**) Quantification of protein levels in the Multi condition relative to those in the Single condition in WT and *naa10*Δ cells. The average fold change ± s.d. was calculated from three biological replicates. Statistical significance was determined by a two-tailed Welch’s *t* test (*p<0.05, **p<0.01). Dashed line represents the same expression level between the Single and Multi conditions. (**D–K**) Same as in (**B, C**), except that shown are Western blots of the RNase P/MRP subunits and their quantification in *naa20*Δ (**D, E**), *naa30*Δ (**F, G**), *naa40*Δ (**H, I**), or *naa50*Δ (**J, K**) cells.

*S*. *cerevisiae* has five NATs: NatA, NatB, NatC, NatD, and NatE whose catalytic subunits are respectively Naa10, Naa20, Naa30, Naa40, and Naa50 [36]. NATs have different sequence specificities for N-acetylation and their substrates can be classified by the first two N-terminal residues except NatD that recognizes approximately five-residue sequence motifs[18, 36, 37]. As shown in Fig 4A, based on the prediction from N-terminal amino acids, the RNase P/MRP subunits can be N-acetylated by NatA, NatB, or NatC. We examined whether the RNase P/MRP subunits are less compensated in *naa10*Δ, *naa20*Δ, *naa30*Δ, *naa40*Δ, or *naa50*Δ cells using the same experimental setup described in Fig 1B. Our experiments showed that Pop4 and Rmp1 in *naa10*Δ (Fig 4B and 4C) and Pop6, Pop8, Rpr2, and Rmp1 in *naa20*Δ (Fig 4D and 4E) were significantly less compensated than those in WT cells, while all tested subunits were compensated in *naa30*Δ or *naa50*Δ as well as in WT cells (Fig 4F, 4G, 4J and 4K). Only Pop3 was uncompensated in *naa40*Δ cells (Fig 4H and 4I), and the half-life of Pop3 was prolonged upon increasing *POP3* copy number in *naa40*Δ cells (S5A and S5B Fig). Of note, Pop3 was stabilized in *naa10*Δ compared to WT cells with or without an increase in gene copy number (S6 Fig) (*p*<0.05, two-tailed Welch’s *t* test), suggesting the involvement of NatA in Pop3 degradation even in unperturbed conditions. We also found that Pop5 compensation was more pronounced in *naa10*Δ than in WT cells (Fig 4B and 4C), suggesting that accelerated Pop5 degradation during dosage compensation is blocked by NatA.

To assess functional redundancy of NATs during dosage compensation, we generated *naa10*Δ *naa20*Δ cells carrying TAP-tagged *POP4* or *RMP1* in the genome. However, this double mutant showed synthetic growth defect (S7A Fig), and we did not observe the colony formation of this double mutant when transformed with multicopy plasmids carrying *POP4* or *RMP1*. We thus measured the amount of Pop4 and Rmp1 without an increase in gene copy number and found that it was not different from that in WT and the single mutants (S7B Fig).

**Fig 5:**
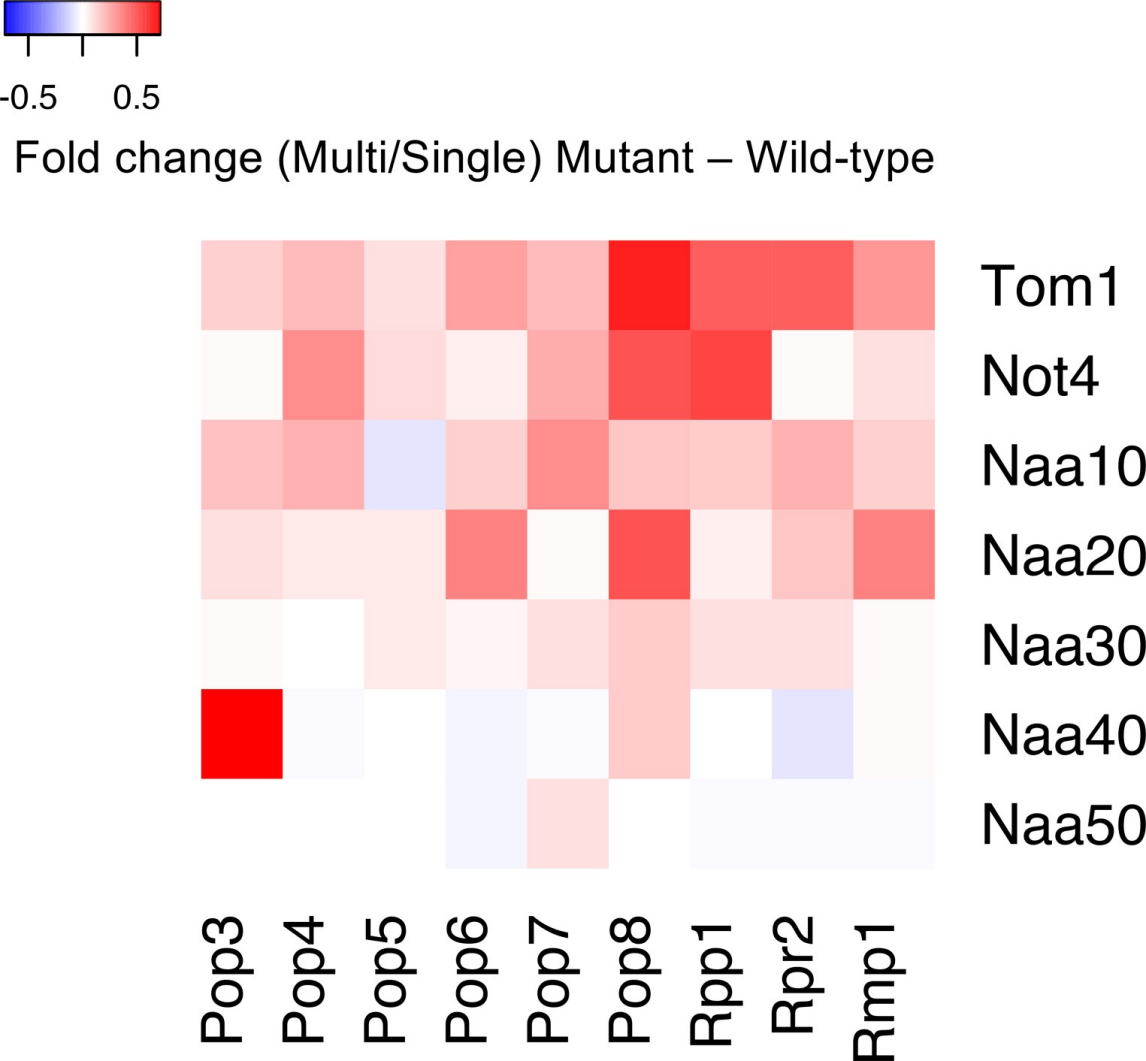
Relative contribution of Tom1, Not4, and NATs to dosage compensation of the RNase P/MRP subunits. The heat map shows a quantitative summary of the contribution of Tom1, Not4, and NATs to dosage compensation of the RNase P/MRP subunits. The basal level is the average fold change in WT cells. The contribution of each factor was calculated as the average fold change in each mutant minus the basal level.

We summarized the contribution of each NAT to the compensation of the RNase P/MRP subunits, leading to several findings (Table 1). First, NatB-mediated compensation of Rmp1 is the only case that can be expected from its N-terminal sequence. Second, the compensation of Pop4 and Pop6 were engaged with NatA and NatB, respectively, although the compensation of Pop4 and Pop6 through NatB and NatC was respectively expected from their sequence specificity for N-acetylation. Third, Rmp1 was compensated through multiple NATs (NatA and NatB). Fourth, the compensation of Pop3 was due in large part to NatD but not NatA. Finally, NATs for the compensation of Pop7 and Rpp1 were not identified. Taken together, the N-terminal sequence cannot simply explain the contribution of NATs to the compensation. Furthermore, NATs-mediated compensation of the RNase P/MRP subunits was mainly mediated by NatA and NatB, although their impact on protein levels was limited (Fig 5). Therefore, these results demonstrate the complexity of the stoichiometry control system that depends only partially on NATs-mediated dosage compensation.

Furthermore, MD-starting proteins Pop4 and Rmp1 were compensated through NatA and Not4, while NatB was also involved in Rmp1 compensation (Figs 2 and 4). Cog1 is also a MD-starting protein that is compensated by NatB and Not4 upon its overexpression [11]. These observations suggest that even though the first two N-terminal residues of substrate proteins are the same, the compensation through the Ac/N-end rule pathway occurs by different combinations of NATs and E3 ligases.

### Canonical NatD substrates, histone H2A/H4, are compensated in the absence of NatD

**Fig 6:**
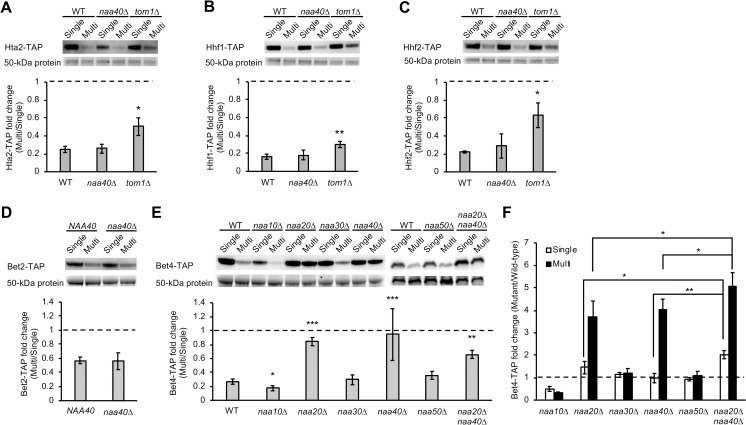
Dissecting the complexity of NATs-mediated dosage compensation by focusing on NatD. (**A–C**) Tom1-mediated dosage compensation of NatD substrates does not require Naa40. Western blot analysis of histone H2A/H4: Hta2-TAP (**A**), Hhf1-TAP (**B**), and Hhf2-TAP (**C**) in WT, *naa40*Δ, or *tom1*Δ cells. The TAP-tagged subunits were detected with PAP. The average fold change ± s.d. was calculated from three biological replicates. (**D**) Western blot analysis of Bet2-TAP in *naa40*Δ cells using PAP. The average fold change ± s.d. was calculated from four biological replicates. (**E**) Western blot analysis of Bet4-TAP in *naa10*Δ, *naa20*Δ, *naa30*Δ, *naa40*Δ, *naa50*Δ, and *naa20*Δ *naa40*Δ cells using PAP. The average fold change ± s.d. was calculated from three biological replicates except the analysis of *naa40*Δ cells in eight biological replicates. Statistical significance was determined by a two-tailed Welch’s *t* test (*p<0.05, **p<0.01, ***p<0.001). Dashed line represents the same expression level between the Single and Multi conditions. (**F**) Comparison of Bet4-TAP levels between WT and each mutant in the Single or Multi conditions. Statistical significance was determined by a one-tailed Welch’s *t* test (*p<0.05, **p<0.01). Dashed line represents the same expression level between WT and each mutant.

As shown in Fig 4H and 4I, NatD efficiently contributes to the compensation of Pop3 that is not identified as a NatD substrate [38]. To further examine whether NATs-mediated dosage compensation is hardly predictable from N-terminal sequence, we next analyzed canonical NatD substrates. NatD is more selective than the other NATs [36], and only histone H2A/H4 subunits are identified as its substrates [38–40]. These histone subunits are MS-starting proteins that can be NatA substrates, but they are N-acetylated in the absence of Naa10 or its auxiliary subunit Naa15 [41]. Thus, we first tested whether NatD is involved in the compensation of histone H2A/H4 subunits: Hta2, Hhf1, and Hhf2. We found that they were subjected to dosage compensation even in the absence of Naa40 (Fig 6A–6C), suggesting that NatD is involved in their N-acetylation but not degradation. Additionally, the compensation was observed not only in *naa40*Δ but also in *naa10*Δ, *naa20*Δ, or *naa30*Δ as well as in WT cells (S8 Fig). We also found that they all were significantly less compensated in *tom1*Δ cells, although the degree of the compensation was different among them (Fig 6A–6C). These results suggest that Tom1-mediated compensation of Hta2, Hhf1, and Hhf2 does not require NATs activity and that N-acetylation and dosage compensation are not necessarily linked. Therefore, dosage compensation of histone H2A/H4 may be mediated by N-acetylation-independent pathway.

### NatD-mediated dosage compensation of a MH-starting protein Bet4

The selectivity of NatD-mediated N-acetylation comes from a sequence motif recognized by NatD and its substrate recognition site tailored for this motif [37]. The N-terminal of Pop3 is SGSL after removal of N-terminal Met by Met-aminopeptidases, which is different from the known motifs involved in histone subunits: SGGK (H2A) and SGRG (H4) [38]. We thus examined whether Bet2, which is a MSGSL-starting protein and a subunit of the Bet2–Bet4 heterodimer [42], is subjected to NatD-mediated compensation. We found that Bet2 was compensated by the same degree in *naa40*Δ and WT cells (Fig 6D), indicating that the first MSGSL is not sufficient to determine the substrates of NatD-mediated compensation. Unexpectedly, Bet4 was remarkably less compensated in *naa40*Δ compared to WT cells (Fig 6E), and indeed, Bet4 was stable upon increasing *BET4* copy number in *naa40*Δ cells due to the prolonged half-life (S5C and S5D Fig). This phenomenon was surprising because Bet4 is a MH-starting protein that is virtually not N-acetylated while N-acetylation of Bet4 was detected but its responsible NATs were not identified [36, 43].

We further examined the contribution of the other NATs to Bet4 compensation and found that Bet4 was significantly less compensated in *naa20*Δ cells (Fig 6E). Thus, NatB and NatD were involved in Bet4 compensation. We also found an increase in Bet4 compensation in *naa10*Δ cells (Fig 6E), suggesting a NatA-mediated block of Bet4 degradation during the compensation. Although these observations are similar to Rmp1 in *naa10*Δ and *naa20*Δ and Pop5 in *naa10*Δ cells (Fig 4B–4E), both effects of NATs deletion were observed only for Bet4. Therefore, Bet4 was identified as the compensated protein whose stoichiometry is bidirectionally controlled by multiple NATs upon genetic perturbations.

To examine whether NatB and NatD control Bet4 level in an additive manner, we analyzed *naa20*Δ *naa40*Δ cells. We observed that Bet4 was compensated in this double mutant to the same degree in *naa20*Δ and *naa40*Δ single mutants (Fig 6E). However, comparison of protein levels between the single and double mutants showed that Bet4 was slightly but significantly more abundant in *naa20*Δ *naa40*Δ cells (Fig 6F and S9 Fig). This small additive effect of the double deletion seems to be consistent with the observation that Bet4 level was close to the uncompensated level even in the single mutants. Because Bet4 was stabilized in the double mutant despite an increase in *BET4* copy number, both NatB and NatD independently contribute to controlling Bet4 level even under unperturbed conditions. These results suggest that their function in the context of Bet4 degradation is not redundant.

## Discussion

To investigate how widespread the Ac/N-end rule pathway explains dosage compensation, we systematically measured the contribution of E3 ubiquitin ligases and NATs to the compensation of the RNase P/MRP subunits (Figs 2 and 4). Our data consisting of 18 subunits and 12 quality control factors, in total 114 combinations, demonstrated that multiple E3 ubiquitin ligases and NATs are involved in the compensation. NATs-mediated compensation was observed for 7 out of 14 tested subunits: 6 of 9 subunits of the RNase P/MRP complexes, none of three histone H2A/H4, and 1 of 2 subunits of the Bet2–Bet4 complex (Figs 4 and 6). However, given that the lack of NATs did not completely reduce the compensation of the tested subunits except for Pop3 in *naa40*Δ and Bet4 in *naa20*Δ and *naa40*Δ cells, stoichiometry control of multiprotein complexes depends only partially on the Ac/N-end rule pathway.

In this study, we manipulated gene copy number to perturb cellular systems and elucidate mechanisms for buffering gene expression perturbations. Our data showed that NATs contribute to proteolysis upon exogenous overexpression of the RNase P/MRP subunits, whereas without overexpression the endogenous protein levels were almost not affected in NATs mutants compared to E3 mutants (Fig 2C and 2F and S6 Fig). This is consistent with a recent comprehensive analysis showing that N-acetylation rarely acts as a degradation signal in physiological conditions in yeast [24]. Therefore, NATs-mediated proteolysis seems to be predominantly triggered upon genetic perturbations. It should be noted that because post-translational dosage compensation is widely used to achieve proper stoichiometry in unperturbed yeast cells [44], physiologically induced stoichiometric imbalance is also fine-tuned by the compensation.

We identified Tom1 as a factor of the stoichiometry control system (Fig 2A and 2B), which is consistent with Tom1-mediated degradation of excess ribosomal subunits [19]. It should be noted that, as discussed in [19], basic isoelectric point is a common characteristic of known Tom1 substrates (Cdc6, Hht2, Yra1, and Rpl26a) and the RNase P/MRP subunits except Pop8 (S10 Fig) [19, 45–47]. The weak electrical interaction between Tom1 with acidic isoelectric point and Pop8 might explain why the compensation degree of Pop8 is lower than the other subunits. Additionally, these substrates as well as Tom1 are nucleic acid binding and localize to the nucleus [48]. These characteristics suggest the possibility that Tom1 tends to compensate nucleic acid binding subunits.

We described the complexity of the stoichiometry control system (Fig 5, Table 1), as (i) multiple E3 ligases Tom1 and Not4 are involved in the compensation of Pop4, Pop5, Pop7, Rpp1, and Rmp1, (ii) N-terminal sequence is not necessarily a determinant of which NATs contribute to dosage compensation, and (iii) multiple NATs are involved in the compensation of Rmp1 and Bet4. Our findings thus suggest that this complexity reflects robustness of the stoichiometry control system. Furthermore, as argued in [49], other mechanisms for sensing unassembled subunits and correcting stoichiometric imbalance may exist. For example, the most recent study showed that protein aggregation of excess subunits functions as the compensation pathway in aneuploid yeast cells [50].

Our observation that NatB and NatD were independently responsible for Bet4 compensation is consistent with the higher substrate specificity of NatD than the other four NATs in yeast (Fig 6F and S9 Fig), although it is unclear whether this is the Bet4-specific molecular phenotype. If NatB could be functionally redundant with NatD, Pop3 should be compensated through NatB in *naa40*Δ and Bet4 should be compensated through NatB in *naa40*Δ and through NatD in *naa20*Δ cells to similar levels in WT cells. Since this is opposite to our results, we speculate that the function of NatB and NatD is not overlapping at least in the context of stoichiometry control. Furthermore, since we observed the stronger impact of deleting *NAA20* than *NAA10* on cell growth and the synthetic growth defect phenotype of *naa10*Δ *naa20*Δ cells (S7 Fig), NatA and NatB may be at least partially involved in the different process. This observation does not eliminate the possibility of functional redundancy but rather suggest additive functions of NatA and NatB. A systematic examination of mutants lacking multiple NATs is required to determine whether there exist overlapping functions between or among NATs and subsequently to unveil more general principles of their functional interactions. One line of support for this possibility comes from the aspect of evolutionary conservation of NATs, as the expression of human NatF (Naa60) increases the fraction of N-acetylated proteins from 68% to 78% in yeast [51], and microscopic analysis of Arl3 whose subcellular localization depends on NatC-mediated N-acetylation demonstrated functional redundancy between yeast NatC and human NatF [52].

The compensation of Pop3 in *naa40*Δ and Bet4 in *naa20*Δ and *naa40*Δ cells was almost fully reduced (Figs 4H, 4I and 6E), supporting the dependency of stoichiometry control on the Ac/N-end rule pathway [11]. However, these are rare cases in our study and the first examples of NatD-mediated dosage compensation, and canonical NatD substrates histone H2A/H4 were compensated even in the absence of Naa40 and the other NATs (Fig 6A–6C, S8 Fig). Importantly, consistent with the stabilization of histone H3 subunit Hht2 by *TOM1* deletion [46], H2A/H4 were less compensated in *tom1*Δ cells. These results suggest that unassembled subunits are selectively degraded by not only the Ac/N-end rule but also other N-acetylation-independent pathways. This is further suggested by the observation that Rpp1 was almost fully uncompensated in *not4*Δ but not in all NATs mutants (Figs 2D, 2E and 4), indicating that Not4-mediated compensation does not necessarily require NATs. Therefore, identifying new compensation E3 ligases is important for better understanding the mechanism of dosage compensation. For example, because a screen using deletion and temperature-sensitive mutants of all non-essential and a few essential E3 ligase genes identified *HEL1*, *HEL2*, *PEP5*, and *SNT2* whose deletion results in defective degradation of excess histone H3/H4 [53], they seem to be candidates for the compensation E3 responsible for nuclear-localized proteins. Indeed, although degradation of excess Rpl26a is mainly mediated by Tom1, Rpl26a level is increased in *pep5*Δ compared to WT cells [19].

We also found that Pop5 and Bet4 were destabilized when they were overexpressed in *naa10*Δ cells (Figs 4B, 4C and 6E). Similarly, previous studies showed that proteins with unacetylated N-terminal Met followed by a small hydrophobic residue and MN-starting proteins are destabilized in *naa30*Δ and *naa20*Δ cells, respectively, by the Arg/N-end rule pathway [24, 54]. However, because N-terminal sequence of Pop5 (MV) and Bet4 (MH) are not appropriate for these cases, their degradation in *naa10*Δ cells might be accelerated by unknown mechanisms. We speculate that the complex stoichiometry is robustly maintained by switching degradation pathways in response to perturbations in the stoichiometry control system. Indeed, the interplay between the Arg/N-end rule and the Ac/N-end rule pathways was proposed based on the observation that the short-lived reporter protein and Msn4 are synergistically stabilized in double mutants *naa20*Δ *ubr1*Δ and *naa30*Δ *ubr1*Δ, respectively [27, 54].

Quality control of multiprotein complexes impacts a broad range of biological processes because of the fact that cellular systems are based on functional complexes. The RNase P and MRP complexes are responsible for maturation of tRNA and rRNA, respectively [28]. Additionally, Pop1, Pop6, and Pop7 are shared with telomerase [55]. Our finding of robust control of dosage balance between Pop6 and Pop7 seems to be in line with their functional importance (Fig 3). It is therefore possible that genome-wide analysis of dosage compensation provides insights into the relationship between the functional importance and essentiality of multiprotein complexes and how strictly the intracellular level of their subunits is controlled. Moreover, the same analysis in NATs mutants leads to a more accurate estimation of how widespread the Ac/N-end rule pathway contributes to stoichiometry control.

More generally, dosage compensation may play a role as a fail-safe mechanism for shaping proteome stoichiometry as discussed above. In agreement with this concept, paralogous complex subunits tend to compensate each other for modulating protein interactome [56, 57]. Furthermore, proteolysis-mediated compensation pervasively buffers physiologically caused stoichiometric imbalance during meiosis [44], which may explain how cells cope with a subset of subunits overexpressed in unperturbed conditions [13]. This finding also suggests that dosage compensation optimizes proteome stoichiometry along the progression of cellular development in each stage of meiosis. Taken together, further dissection of the complexity and working principles of the stoichiometry control system will help to understand buffering mechanism for proteome concentration against both intrinsic and extrinsic genetic perturbations.

## Materials and methods

### Strains, plasmids, and media

For construction of the single deletion strains lacking a gene encoding the E3 ubiquitin ligase (*TOM1*, *NOT4*, *DOA10*, *LTN1*, or *HEL2*) or N-acetyltransferase (*NAA10*, *NAA20*, *NAA30*, *NAA40*, or *NAA50*) and carrying a TAP-tagged gene of interest, the deletion collection and the TAP collection of haploid yeast (Dharmacon) were used. BY4741 carrying Pop4-TAP, Rmp1-TAP, or Bet4-TAP were constructed in this study. Genomic DNA of each single deletion strain was extracted, and then, each locus replaced with the *kanMX4* cassette was amplified by PCR with a primer library prepared in our previous study [5]. For construction of the double deletion strains (*naa10*Δ *naa20*Δ and *naa20*Δ *naa40*Δ), the *hphNT1* cassette amplified from pFA6-hphNT1 plasmid was used for replacing *NAA20* locus in the genome of *naa10*Δ carrying Pop4-TAP or Rmp1-TAP and *naa40*Δ carrying Bet4-TAP, respectively. The TAP-tagged strains were transformed with the PCR products by the lithium acetate method and selected on YPD plates containing G418 (200 μg/mL) or HygB (300 μg/mL). For construction of plasmids, DNA fragment of each target region was amplified from the genome and cloned into pTOW40836 by homologous recombination in BY4741 cells. The TAP-tagged strains described above were transformed with the plasmids and selected on SC medium lacking uracil.

### Measurement of plasmid copy number

The plasmid copy number was measured by the gTOW technique [58]. The cells grown at log-phase for Western blotting were harvested for the gTOW analysis from the same culture. In short, 200 μL of the culture was centrifuged and the pelleted cells were suspended in 100 μL of zymolyase solution [2.5 mg/mL Zymolyase-100T dissolved in 1.2 M sorbitol, 10 mM sodium phosphate (pH7.5)] and incubated at 37°C for 15 min for DNA extraction. The suspension was heated at 100°C for 10 min, cooled at –80°C for 10 min, and again heated at 100°C for 10 min. After cooling down to room temperature (RT) followed by centrifugation, the supernatant was subjected to real-time quantitative PCR with Lightcycler 480 (Roche). The primers for *LEU3* in the genome or *leu2d* gene in pTOW40836 and SYBR Green I Master (Roche) were used for PCR. The resulting plasmid copy number was calculated based on the expression levels of *LEU3* and *leu2d* genes according to the method described previously [58].

### Western blot analysis

Cells were grown at 30°C in 2 mL of SC–Ura medium for overnight and then measured the optical density at 600 nm (OD_600_) and inoculated into fresh medium at initial OD_600_ of 0.5 in 3 mL. After 4 h, 1 OD_600_ units were harvested at log-phase when OD_600_ was around 1. The cells were treated with 1 mL of 0.2 N NaOH for 5 min at RT and centrifuged at 15,000 rpm for 1 min. The pelleted cells were suspended in 50 μL of 1× NuPAGE LDS Sample Buffer (Invitrogen) containing 100 mM DTT and heated at 100°C for 5 min. For the analysis of the TAP-tagged proteins, two-fold serially diluted lysates were analyzed as described below, and we confirmed that 0.025 OD_600_ units provide appropriate signal in the linear range. The extract diluted 8-fold with 1× NuPAGE LDS Sample Buffer, corresponding to 0.025 OD_600_ units, was separated by polyacrylamide gel electrophoresis with lithium dodecyl sulfate (SDS-PAGE) on NuPAGE 4%–12% Bis-Tris Gel (Invitrogen). For the analysis of the GFP-tagged proteins, 0.2 OD_600_ units were subjected to SDS-PAGE. The separated proteins were transferred onto PVDF membrane using the iBlot Transfer Stack PVDF membrane (Invitrogen). The blotted membrane was treated with PBST [1× PBS, 0.1% Tween 20] for 10 min, and then blocked with 4% skim milk in PBST for 1 h at RT. The TAP-tagged proteins were probed with PAP (Sigma-Aldrich) (1:2,000) for 1 h at RT. The GFP-tagged proteins were probed with anti-GFP antibody (Roche) (1:1,000) and peroxidase-conjugated secondary antibody (Nichirei Biosciences) (1:1,000) for 1 h at RT. After washing the membrane with PBST for 5 min for three times, chemiluminescence was induced by adding 500 μL of SuperSignal West Femto Maximum Sensitivity Substrate (Thermo Scientific) on the membrane and detected using LAS-4000 image analyzer (Fujifilm) and ImageQuant LAS 4000 (GE Healthcare). Quantification of the band intensity was carried out using ImageQuant TL (GE Healthcare) and the fold change was calculated after background subtraction. After washing the membrane with sterile water for 5 min for three times, total proteins were visualized by Coomassie staining with SimplyBlue SafeStain (Invitrogen) to confirm equal loading of proteins. The stained membrane was digitized using LAS-4000 image analyzer and ImageQuant LAS 4000. Representative blots and corresponding Coomassie stains are shown in S11 Fig.

For the analysis of histone H2A/H4 and the Bet2–Bet4 heterodimer, the harvested cells were treated with 1 mL of 0.2 N NaOH for 5 min at RT. Cells were suspended in 1× NuPAGE LDS Sample Buffer (Invitrogen) and then heated at 70°C for 10 min. The supernatant corresponding to 0.5 OD_600_ units was labeled with EzLabel FluoroNeo (ATTO) and subjected to SDS-PAGE, followed by Western blot with PAP (Sigma-Aldrich) (1:2,000) as described above.

### Cycloheximide chase assay

The degradation rate of Pop3-TAP and Bet4-TAP was measured as previously described [7]. Briefly, the log-phase culture corresponding to 1 OD_600_ unit of cells was harvested for time point 0 and afterwards CHX was added to a final concentration of 200 μg/mL. Cells were harvested after 1, 2, 4, and 6.5 h, followed by total protein extraction in 1× NuPAGE LDS Sample Buffer. The supernatant corresponding to 0.5 OD_600_ units was subjected to Western blot analysis using PAP as described above. The remaining protein level at each time point was calculated against that at time point 0.

## Supporting information

S1 Fig Identification of E3 ubiquitin ligases involved in dosage compensation. (**A**) An experimental setup for the screen of E3 ubiquitin ligases involved in dosage compensation. The dosage-compensated proteins tagged with green fluorescent protein (GFP) were expressed from multicopy plasmid pTOW40836 containing the native regulatory sequences, including promoter and 5´ and 3´ untranslated regions. If the tested E3 ligase is not responsible for degradation of the target protein, the protein level is the same between WT and E3 mutant cells (left and middle panels). On the other hand, if the target protein is degraded through the tested E3 ligase, the protein level increases in the E3 mutant compared to WT cells (right and left panels). (**B**) Western blot of the GFP-tagged dosage-compensated proteins in E3 mutants using anti-GFP antibody. Coomassie staining of a 50-kDa protein, corresponding to enolase, is shown as a loading control. (**C**) Gene copy number during dosage compensation. Western blot detected the increased amount of Pop5 in *tom1*Δ and *not4*Δ and Saw1 in *doa10*Δ compared to those in WT cells, although the plasmid copy number was almost the same among the tested strains. Thus, Tom1 and Not4 and Doa10 were identified as E3 ubiquitin ligases involved in degradation of Pop5 and Saw1, respectively. Bar graph represents the copy numbers of pTOW40836 carrying each of the indicated genes in WT or E3 mutants. The average copy numbers ± s.d. were calculated from four technical replicates.
(TIF)

S2 Fig Quantification of Western blot data. (**A, B**) Coomassie stained PVDF membrane (**A**) after Western blotting with PAP (**B**) of two-fold serially diluted cell lysates prepared from *tom1*Δ Pop3-TAP or Pop8-TAP cells cultured in the Single condition. (**C**) Quantification of the area of a 50-kDa protein in (**A**). The signal intensity of each band was measured after background subtraction, and the net intensity was plotted on the y-axis. The amount of lysate from *tom1*Δ Pop3-TAP and Pop8-TAP had a correlation coefficient (*R*^*2*^) of 0.99 and 0.97 with the net intensity, respectively. (**D**) Quantification of the area of Pop3-TAP and Pop8-TAP in (**B**). The signal intensity of each band was measured after background subtraction, and the net intensity was plotted on the y-axis. The amount of lysate from *tom1*Δ Pop3-TAP and Pop8-TAP had *R*^*2*^ of 0.99 and 0.99 with the net intensity, respectively. (**E**) Comparison of protein levels between the Single and Multi conditions in WT or each mutant. Shown as an example is Pop4-TAP in WT and *tom1*Δ cells. The band intensity of Pop4-TAP in the Multi condition was divided by that in the Single condition in each strain. The full Coomassie band on the PVDF membrane was used for normalization. (**F**) Comparison of protein levels between WT and each mutant in the Single or Multi conditions. The band intensity of Pop4-TAP in *tom1*Δ was divided by that in WT cells in each copy number condition. Data are from Fig 2A–2C.
(TIF)

S3 Fig Western blot analysis of the RNase P/MRP subunits in *doa10*Δ cells. (**A–C**) Doa10 is not involved in dosage compensation of the RNase P/MRP subunits. Western blot analysis of Pop3-TAP in two biological replicates (**A**) and the other RNase P/MRP subunits except for Pop5 (**B, C**) using PAP in *doa10*Δ cells. Dashed line represents the same expression level between the Single and Multi conditions.
(TIF)

S4 Fig Western blot analysis of Rmp1-TAP in RQC mutants. Western blot analysis of Rmp1-TAP using PAP in *hel2*Δ and *ltn1*Δ cells. Shown images are from the same membrane.
(TIF)

S5 Fig CHX chase experiments of Pop3-TAP and Bet4-TAP in *naa40*Δ cells. (**A, B**) CHX chase experiments of Pop3-TAP in WT and *naa40*Δ cells. Western blot with PAP and SDS-PAGE of a 50-kDa protein as a loading control (**A**). Quantification of Pop3-TAP levels in the Single (left) or Multi (right) conditions (**B**). The average protein level ± s.d. was calculated from three biological replicates. (**C, D**) Same as in (**A, B**), except that shown are Western blot and quantification of Bet4-TAP.
(TIF)

S6 Fig The effect of NATs on the endogenous protein level of the RNase P/MRP subunits. (**A–E**) Comparison of protein levels between WT and *naa10*Δ (**A**), *naa20*Δ (**B**), *naa30*Δ (**C**), *naa40*Δ (**D**), or *naa50*Δ (**E**) cells in the Single or Multi conditions. The average fold change ± s.d. was calculated from three biological replicates. Dashed line represents the same expression level between WT and mutant cells. Data are from Fig 4B–4K.
(TIF)

S7 Fig Synthetic growth defect of *naa10*Δ *naa20*Δ cells. (**A**) Spot test of *naa10*Δ *naa20*Δ cells. Overnight cultures of indicated strains in YPD medium diluted to 0.1 OD_600_ and its 10-fold serial dilutions were spotted on YPD plates and incubated at 30°C for 6 days. (**B**) Western blots of Pop4-TAP (left) and Rmp1-TAP (right) in *naa10*Δ *naa20*Δ double mutant. All strains were cultured in YPD medium due to no colony formation of *naa10*Δ *naa20*Δ cells transformed with multicopy plasmids pTOW40836 carrying *POP4* or *RMP1* on SC–Ura plate. Quantification from three biological replicates is shown below.
(TIF)

S8 Fig Dosage compensation of Hhf2 in NATs mutants. Western blot analysis of Hhf2-TAP in WT, *naa10*Δ, *naa20*Δ, *naa30*Δ, and *naa40*Δ cells. Hhf2-TAP was detected with PAP (top) and quantified (bottom). Dashed line represents the same expression level between the Single and Multi conditions.
(TIF)

S9 Fig Bet4 was slightly but significantly more abundant in *naa20*Δ *naa40*Δ cells. (**A, B**) Higher Bet4-TAP level in *naa20*Δ *naa40*Δ double mutant compared to the single mutants in the Single (**A**) and Multi (**B**) conditions. Quantification of three biological replicates is shown. Data are from Fig 6F.
(TIF)

S10 Fig The isoelectric point of the RNase P/MRP subunits and Tom1 substrates. The isoelectric point of Tom1 is 4.8, while that of the RNase P/MRP subunits, except Pop8, and known Tom1 substrates is around 10.
(TIF)

S11 Fig Representative blots and corresponding Coomassie stains of Western blots of the RNase P/MRP subunits in E3 ligases or NATs mutants. **(A–G)** Shown are full length blots and Coomassie stains used for the analysis of *tom1*Δ (**A**), *not4*Δ (**B**), *naa10*Δ (**C**), *naa20*Δ (**D**), *naa30*Δ (**E**), *naa40*Δ (**F**), or *naa50*Δ (**G**) mutants, corresponding to Figs 2A, 2D, 4B, 4D, 4F, 4H and 4J.
(TIF)
