# Supplementary figures and images for: Exploring the Complexity of Protein-Level Dosage Compensation that Fine-Tunes Stoichiometry of Multiprotein Complexes
Source: PLoS Genet. 2020 Oct 28;16(10):e1009091. doi: 10.1371/journal.pgen.1009091 (PMC7652333; doi:10.1371/journal.pgen.1009091)

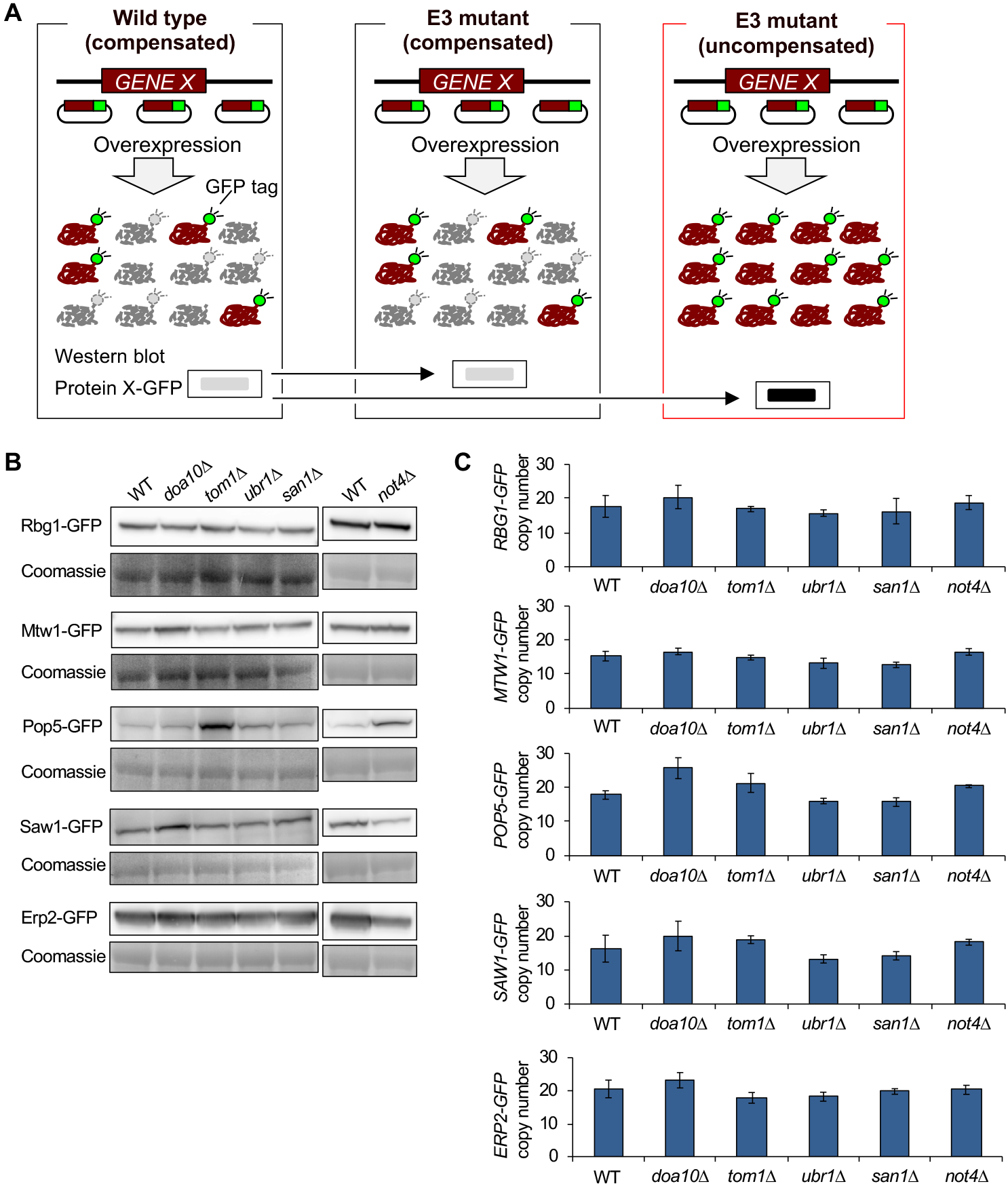

Supplement: S1 Fig — (A) An experimental setup for the screen of E3 ubiquitin ligases involved in dosage compensation. The dosage-compensated proteins tagged with green fluorescent protein (GFP) were expressed from multicopy plasmid pTOW40836 containing the native regulatory sequences, including promoter and 5´ and 3´ untranslated regions. If the tested E3 ligase is not responsible for degradation of the target protein, the protein level is the same between WT and E3 mutant cells (left and middle panels). On the other hand, if the target protein is degraded through the tested E3 ligase, the protein level increases in the E3 mutant compared to WT cells (right and left panels). (B) Western blot of the GFP-tagged dosage-compensated proteins in E3 mutants using anti-GFP antibody. Coomassie staining of a 50-kDa protein, corresponding to enolase, is shown as a loading control. (C) Gene copy number during dosage compensation. Western blot detected the increased amount of Pop5 in tom1Δ and not4Δ and Saw1 in doa10Δ compared to those in WT cells, although the plasmid copy number was almost the same among the tested strains. Thus, Tom1 and Not4 and Doa10 were identified as E3 ubiquitin ligases involved in degradation of Pop5 and Saw1, respectively. Bar graph represents the copy numbers of pTOW40836 carrying each of the indicated genes in WT or E3 mutants. The average copy numbers ± s.d. were calculated from four technical replicates. (TIF) [file pgen.1009091.s001.tif]

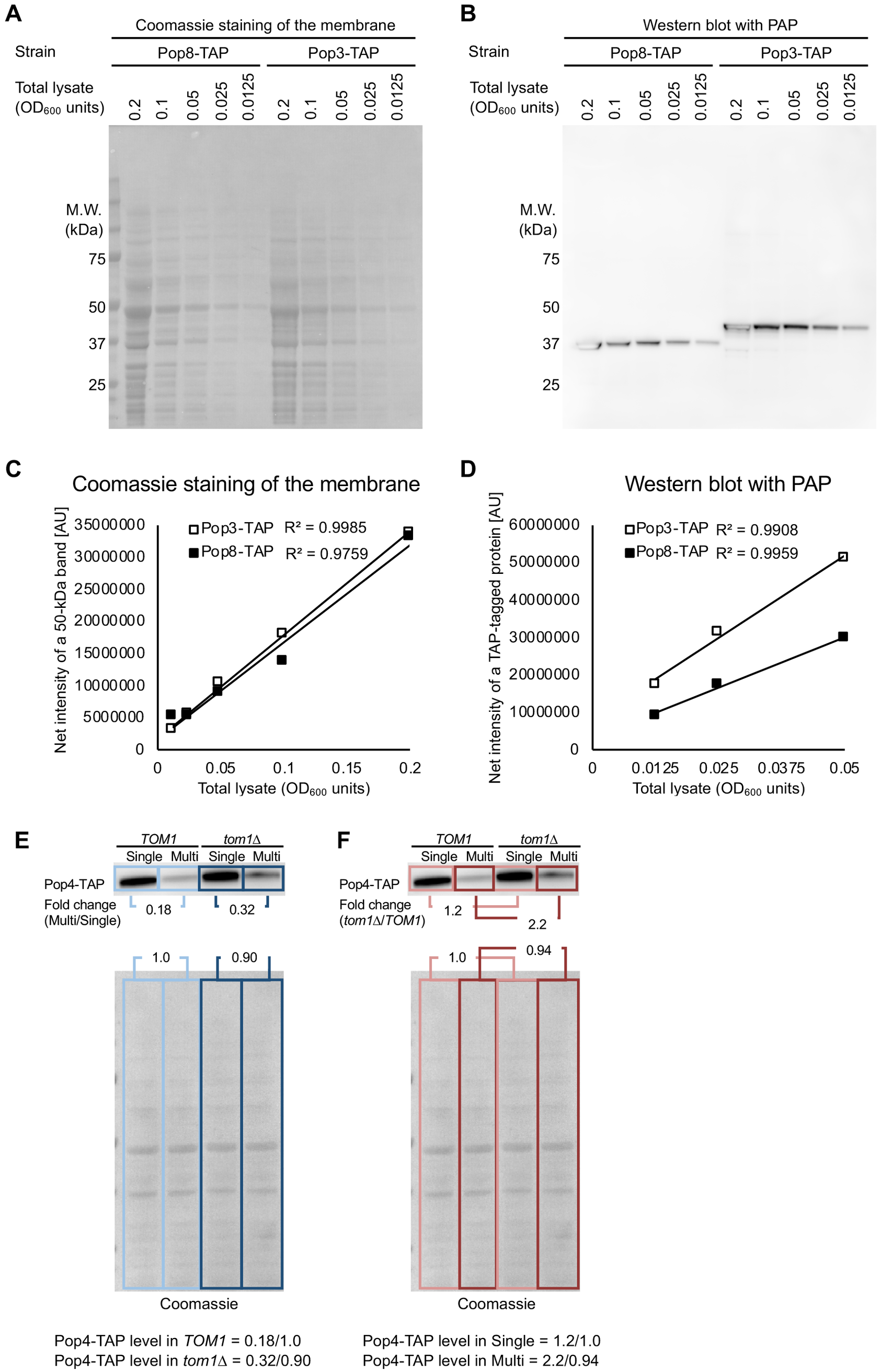

Supplement: S2 Fig — (A, B) Coomassie stained PVDF membrane (A) after Western blotting with PAP (B) of two-fold serially diluted cell lysates prepared from tom1Δ Pop3-TAP or Pop8-TAP cells cultured in the Single condition. (C) Quantification of the area of a 50-kDa protein in (A). The signal intensity of each band was measured after background subtraction, and the net intensity was plotted on the y-axis. The amount of lysate from tom1Δ Pop3-TAP and Pop8-TAP had a correlation coefficient (R2) of 0.99 and 0.97 with the net intensity, respectively. (D) Quantification of the area of Pop3-TAP and Pop8-TAP in (B). The signal intensity of each band was measured after background subtraction, and the net intensity was plotted on the y-axis. The amount of lysate from tom1Δ Pop3-TAP and Pop8-TAP had R2 of 0.99 and 0.99 with the net intensity, respectively. (E) Comparison of protein levels between the Single and Multi conditions in WT or each mutant. Shown as an example is Pop4-TAP in WT and tom1Δ cells. The band intensity of Pop4-TAP in the Multi condition was divided by that in the Single condition in each strain. The full Coomassie band on the PVDF membrane was used for normalization. (F) Comparison of protein levels between WT and each mutant in the Single or Multi conditions. The band intensity of Pop4-TAP in tom1Δ was divided by that in WT cells in each copy number condition. Data are from Fig 2A–2C. (TIF) [file pgen.1009091.s002.tif]

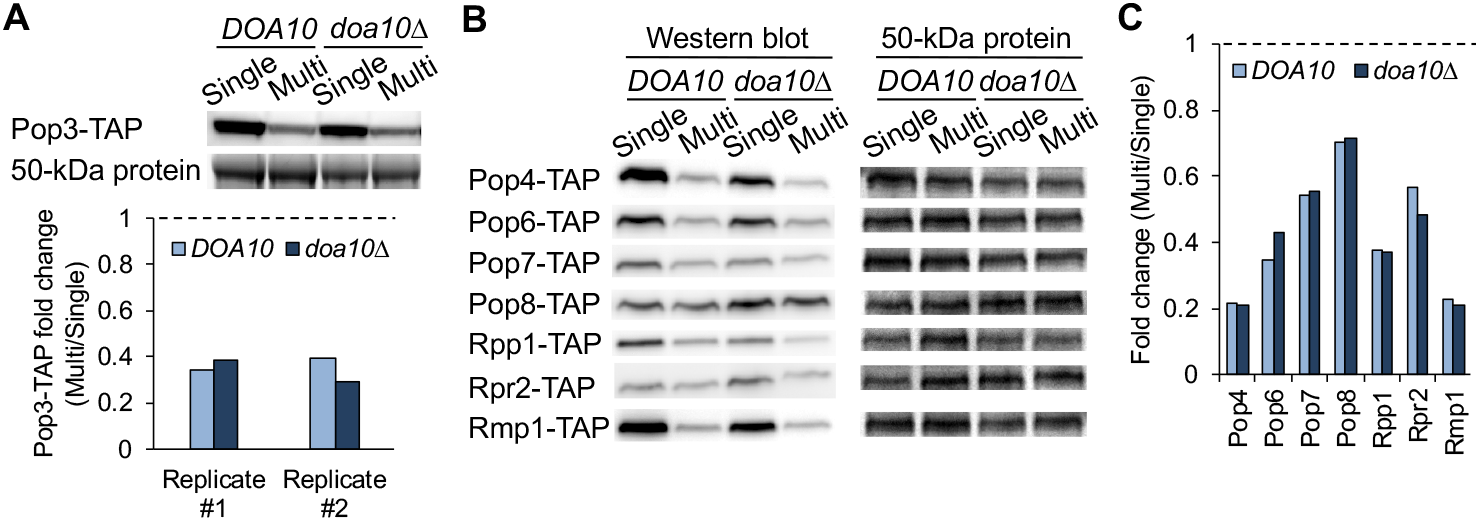

Supplement: S3 Fig — (A–C) Doa10 is not involved in dosage compensation of the RNase P/MRP subunits. Western blot analysis of Pop3-TAP in two biological replicates (A) and the other RNase P/MRP subunits except for Pop5 (B, C) using PAP in doa10Δ cells. Dashed line represents the same expression level between the Single and Multi conditions. (TIF) [file pgen.1009091.s003.tif]

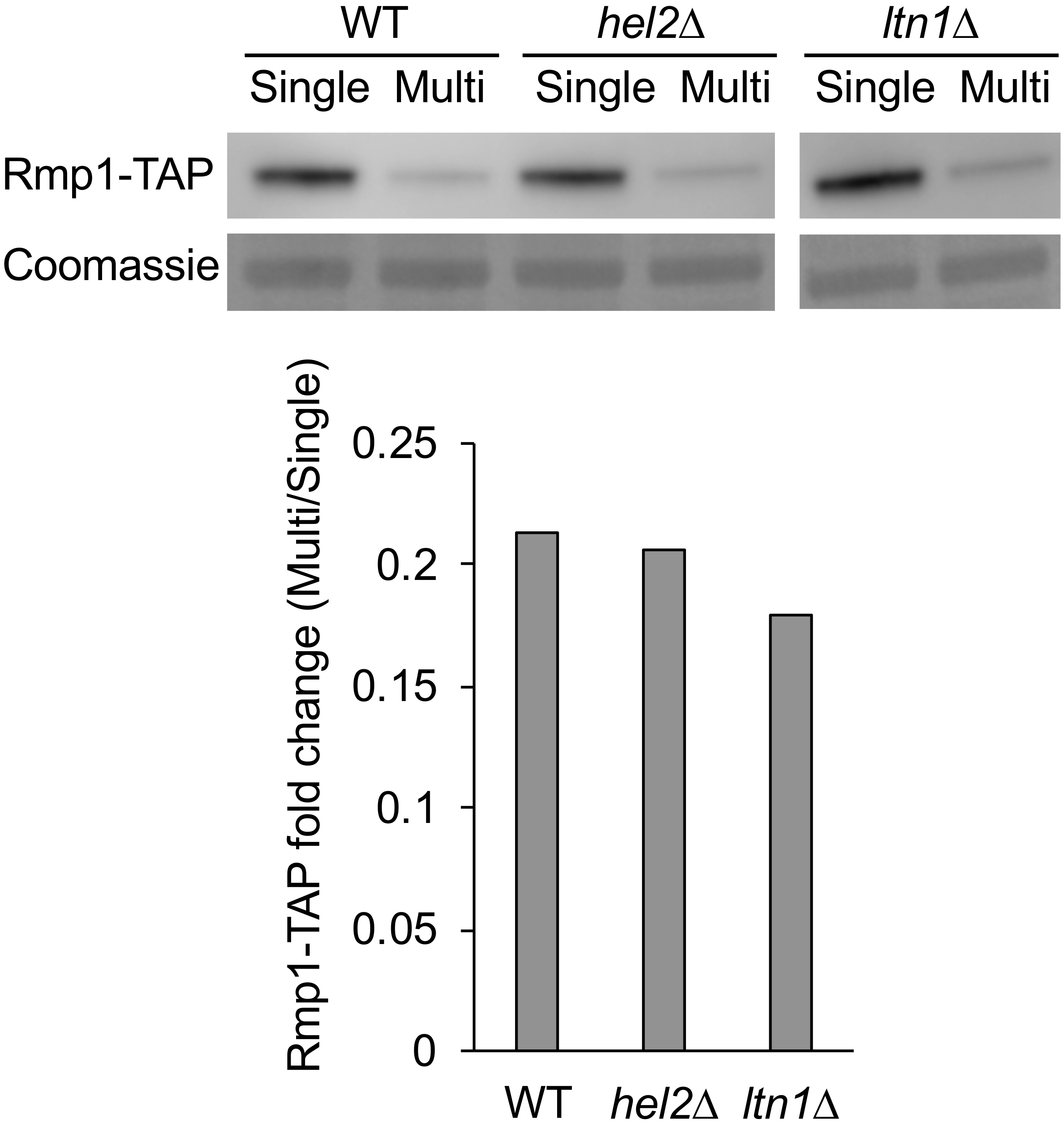

Supplement: S4 Fig — Western blot analysis of Rmp1-TAP using PAP in hel2Δ and ltn1Δ cells. Shown images are from the same membrane. (TIF) [file pgen.1009091.s004.tif]

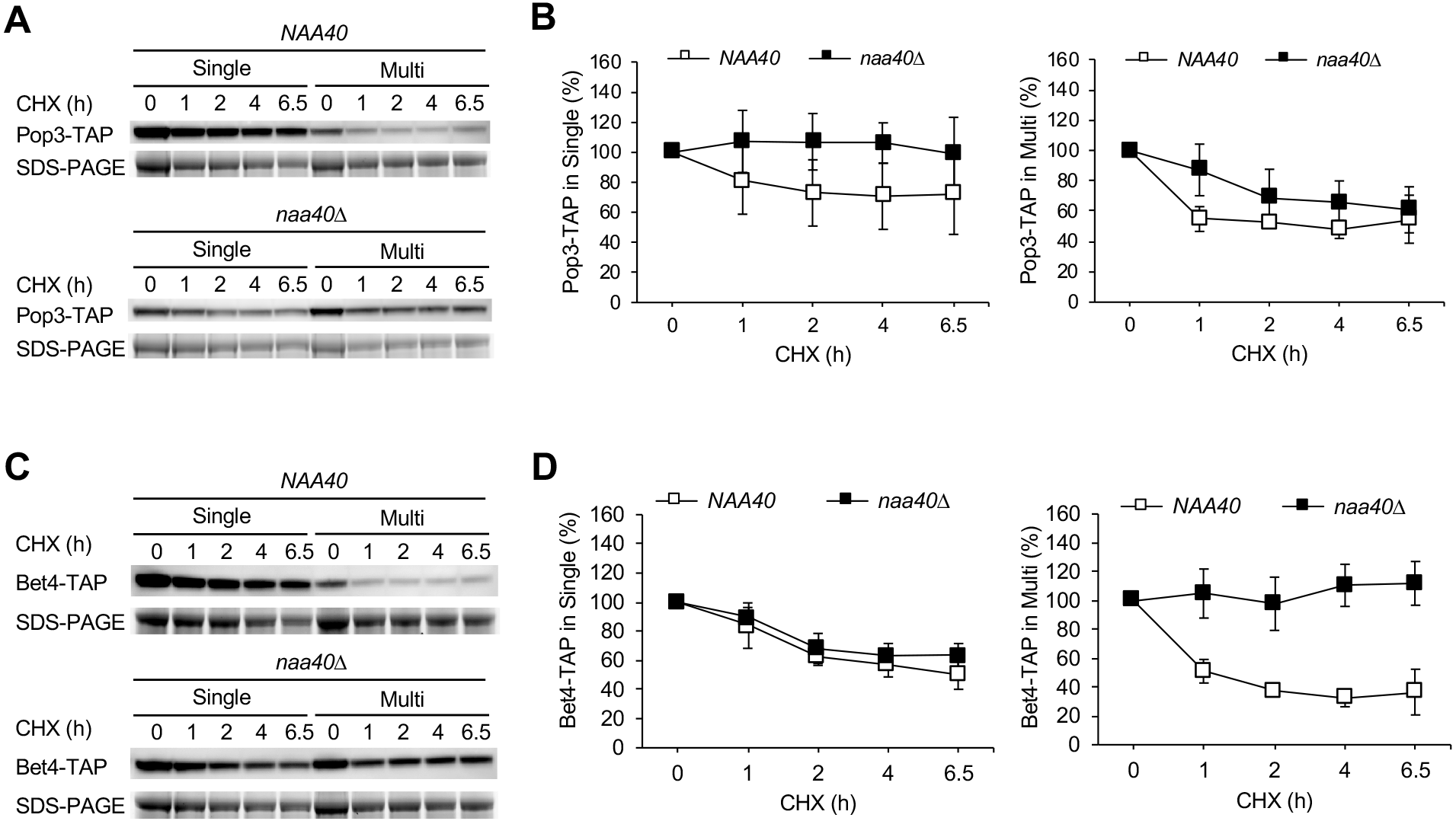

Supplement: S5 Fig — (A, B) CHX chase experiments of Pop3-TAP in WT and naa40Δ cells. Western blot with PAP and SDS-PAGE of a 50-kDa protein as a loading control (A). Quantification of Pop3-TAP levels in the Single (left) or Multi (right) conditions (B). The average protein level ± s.d. was calculated from three biological replicates. (C, D) Same as in (A, B), except that shown are Western blot and quantification of Bet4-TAP. (TIF) [file pgen.1009091.s005.tif]

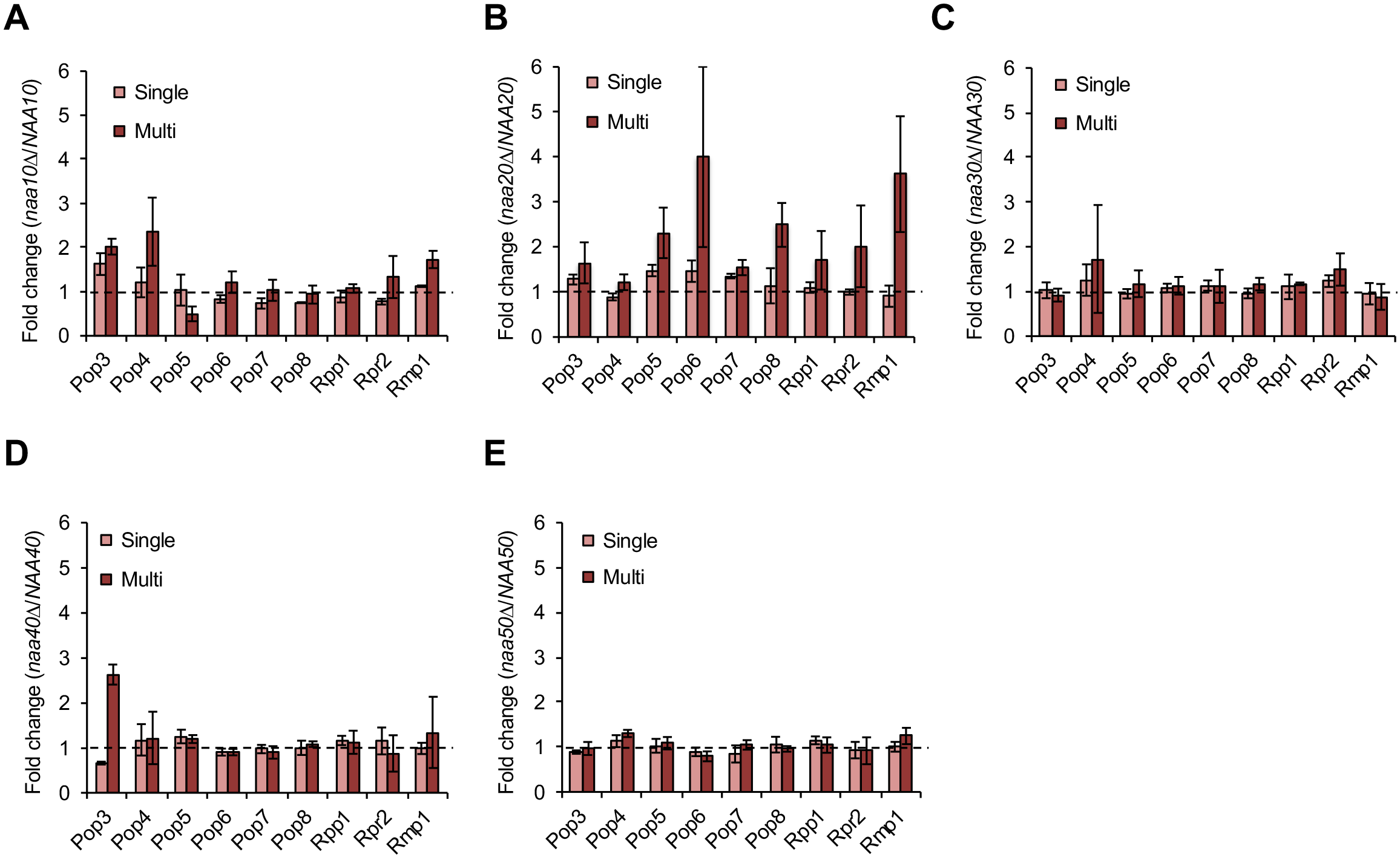

Supplement: S6 Fig — (A–E) Comparison of protein levels between WT and naa10Δ (A), naa20Δ (B), naa30Δ (C), naa40Δ (D), or naa50Δ (E) cells in the Single or Multi conditions. The average fold change ± s.d. was calculated from three biological replicates. Dashed line represents the same expression level between WT and mutant cells. Data are from Fig 4B–4K. (TIF) [file pgen.1009091.s006.tif]

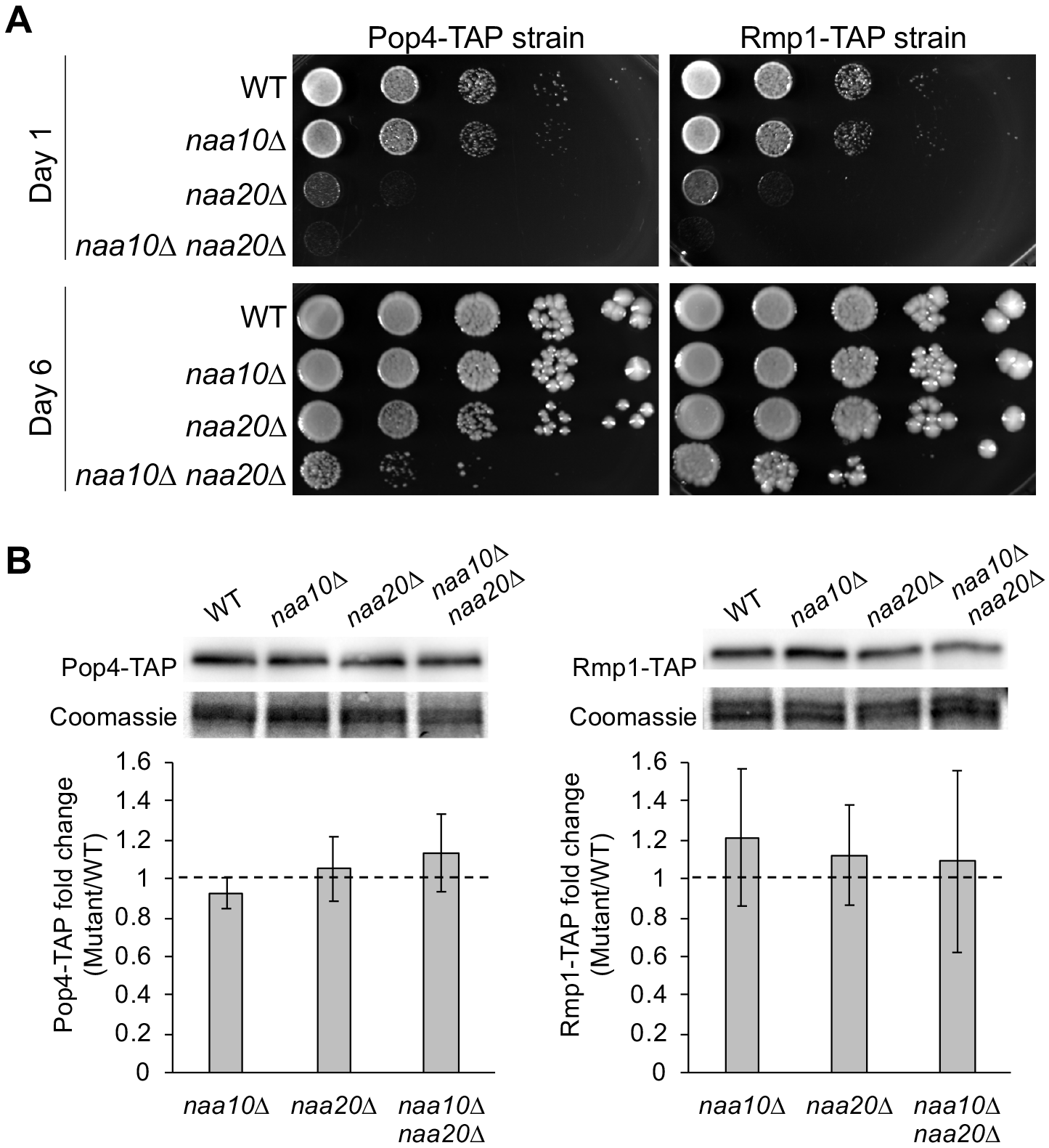

Supplement: S7 Fig — (A) Spot test of naa10Δ naa20Δ cells. Overnight cultures of indicated strains in YPD medium diluted to 0.1 OD600 and its 10-fold serial dilutions were spotted on YPD plates and incubated at 30°C for 6 days. (B) Western blots of Pop4-TAP (left) and Rmp1-TAP (right) in naa10Δ naa20Δ double mutant. All strains were cultured in YPD medium due to no colony formation of naa10Δ naa20Δ cells transformed with multicopy plasmids pTOW40836 carrying POP4 or RMP1 on SC–Ura plate. Quantification from three biological replicates is shown below. (TIF) [file pgen.1009091.s007.tif]

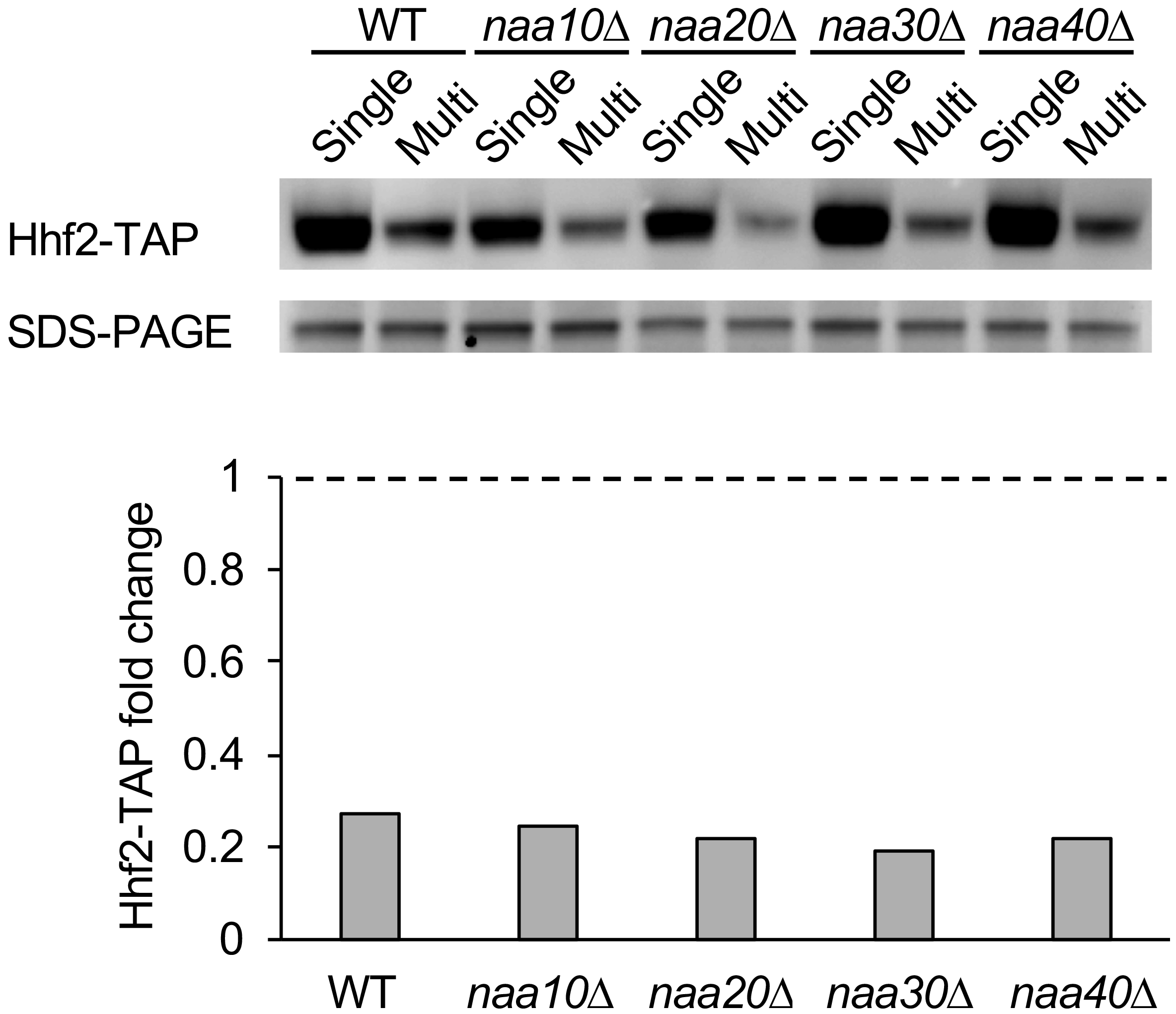

Supplement: S8 Fig — Western blot analysis of Hhf2-TAP in WT, naa10Δ, naa20Δ, naa30Δ, and naa40Δ cells. Hhf2-TAP was detected with PAP (top) and quantified (bottom). Dashed line represents the same expression level between the Single and Multi conditions. (TIF) [file pgen.1009091.s008.tif]

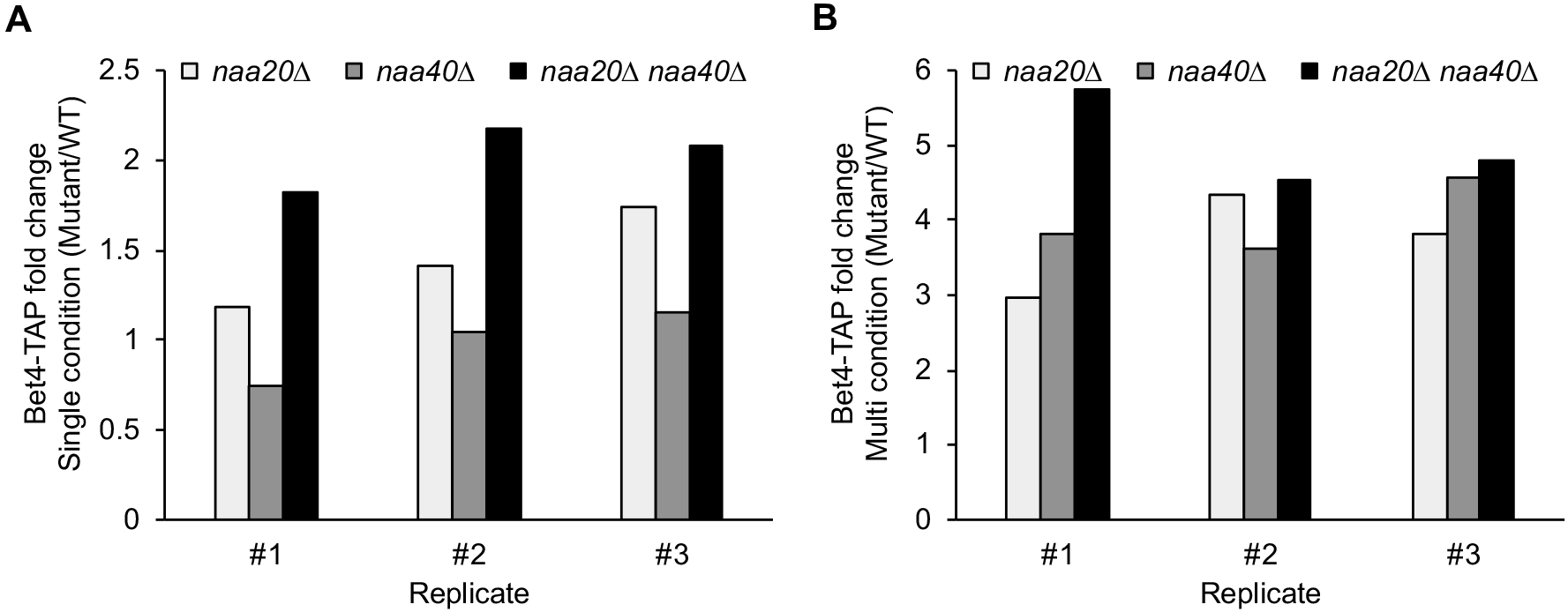

Supplement: S9 Fig — (A, B) Higher Bet4-TAP level in naa20Δ naa40Δ double mutant compared to the single mutants in the Single (A) and Multi (B) conditions. Quantification of three biological replicates is shown. Data are from Fig 6F. (TIF) [file pgen.1009091.s009.tif]

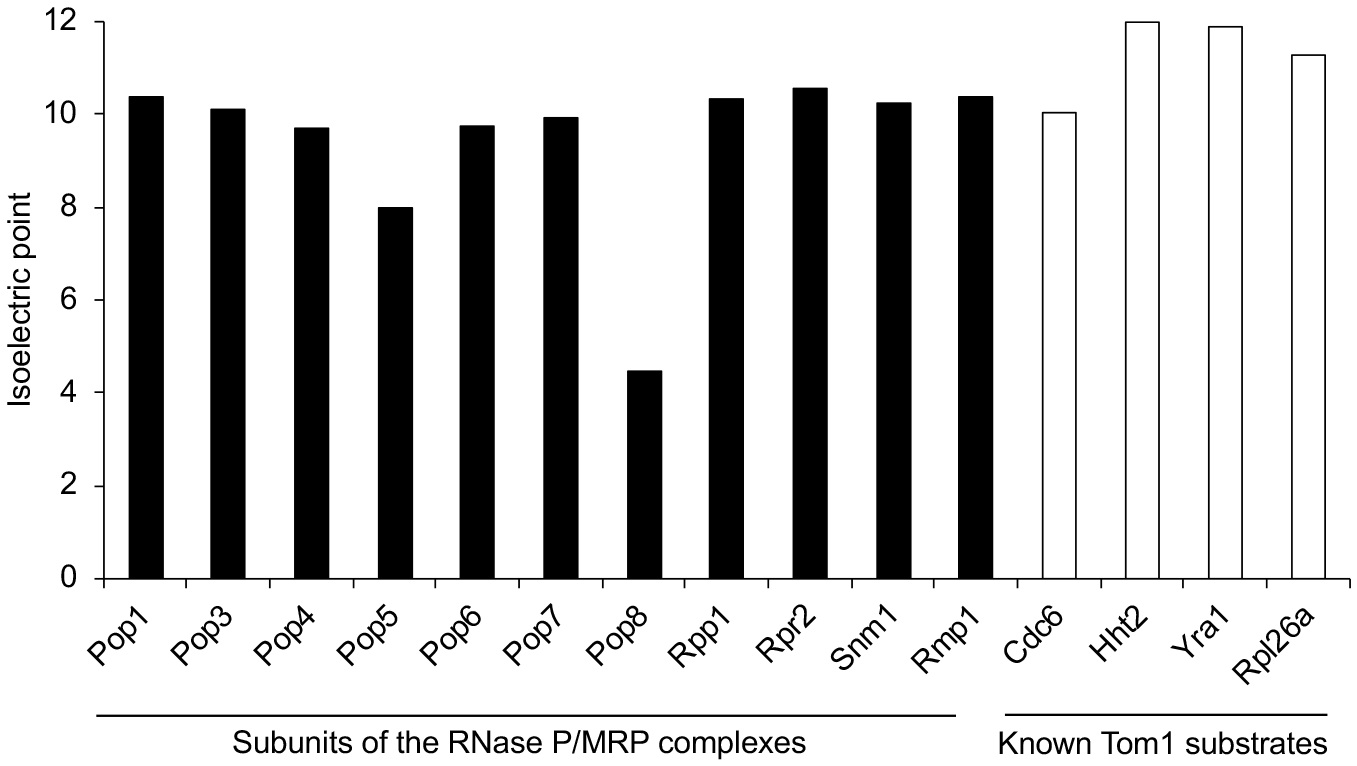

Supplement: S10 Fig — The isoelectric point of Tom1 is 4.8, while that of the RNase P/MRP subunits, except Pop8, and known Tom1 substrates is around 10. (TIF) [file pgen.1009091.s010.tif]

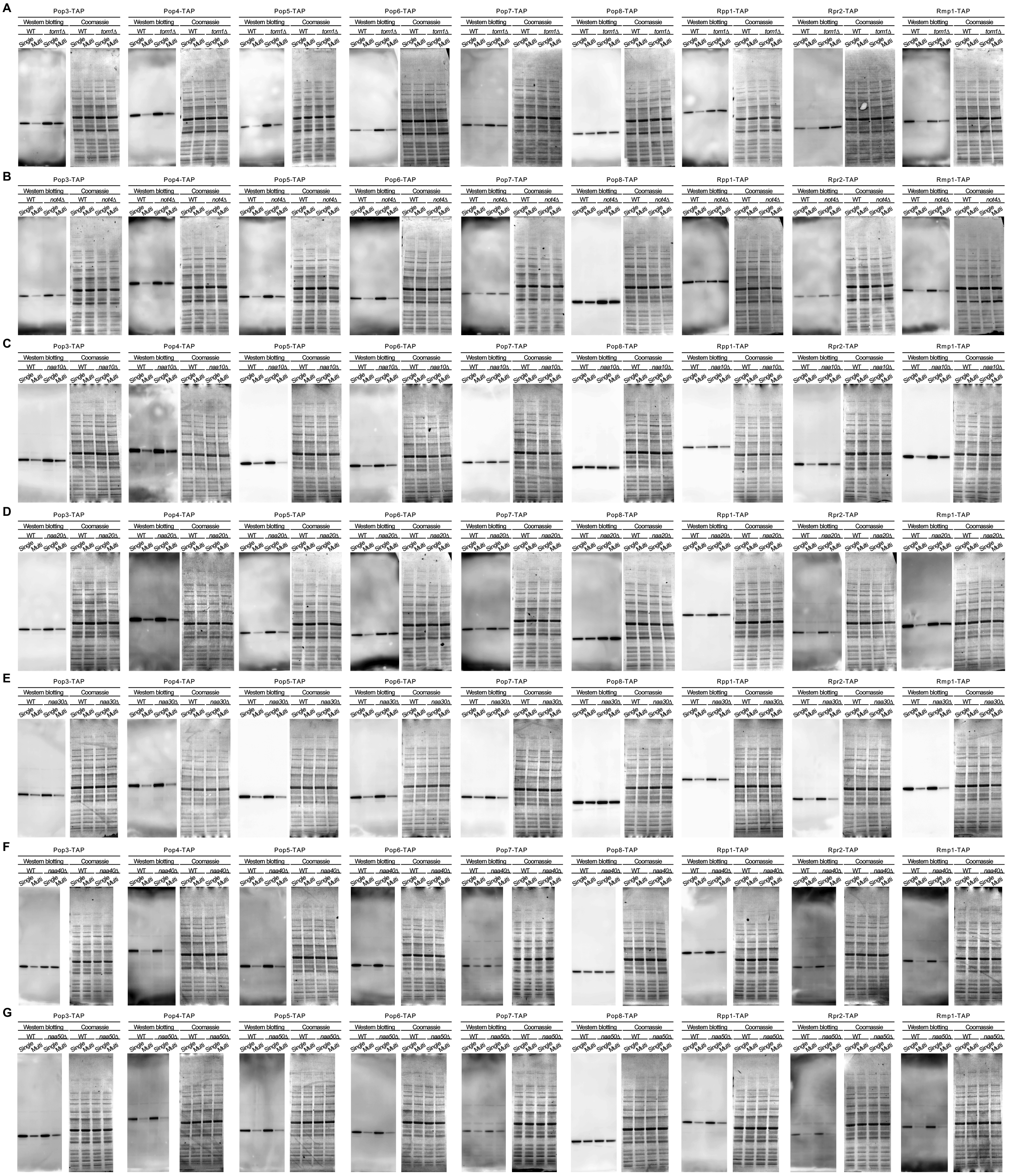

Supplement: S11 Fig — (A–G) Shown are full length blots and Coomassie stains used for the analysis of tom1Δ (A), not4Δ (B), naa10Δ (C), naa20Δ (D), naa30Δ (E), naa40Δ (F), or naa50Δ (G) mutants, corresponding to Figs 2A, 2D, 4B, 4D, 4F, 4H and 4J. (TIF) [file pgen.1009091.s011.tif]
